# Systematic experimental study of quantum interference effects in anthraquinoid molecular wires†

**DOI:** 10.1039/c8na00223a

**Published:** 2019-02-07

**Authors:** Marco Carlotti, Saurabh Soni, Xinkai Qiu, Eric Sauter, Michael Zharnikov, Ryan C. Chiechi

**Affiliations:** Zernike Institute for Advanced Materials Nijenborgh 4 9747 AG Groningen The Netherlands r.c.chiechi@rug.nl; Stratingh Institute for Chemistry, University of Groningen Nijenborgh 4 9747 AG Groningen The Netherlands; Applied Physical Chemistry, Heidelberg University Im Neuenheier Feld 253 Heidelberg 69120 Germany

## Abstract

In order to translate molecular properties in molecular-electronic devices, it is necessary to create design principles that can be used to achieve better structure–function control oriented toward device fabrication. In molecular tunneling junctions, cross-conjugation tends to give rise to destructive quantum interference effects that can be tuned by changing the electronic properties of the molecules. We performed a systematic study of the tunneling charge-transport properties of a series of compounds characterized by an identical cross-conjugated anthraquinoid molecular skeleton but bearing different substituents at the 9 and 10 positions that affect the energies and localization of their frontier orbitals. We compared the experimental results across three different experimental platforms in both single-molecule and large-area junctions and found a general agreement. Combined with theoretical models, these results separate the intrinsic properties of the molecules from platform-specific effects. This work is a step towards explicit synthetic control over tunneling charge transport targeted at specific functionality in (proto-)devices.

## Abstract 2

In order to translate molecular properties in molecular-electronic devices, it is necessary to create design principles that can be used to achieve better structure–function control oriented toward device fabrication.

## Graphical abstract

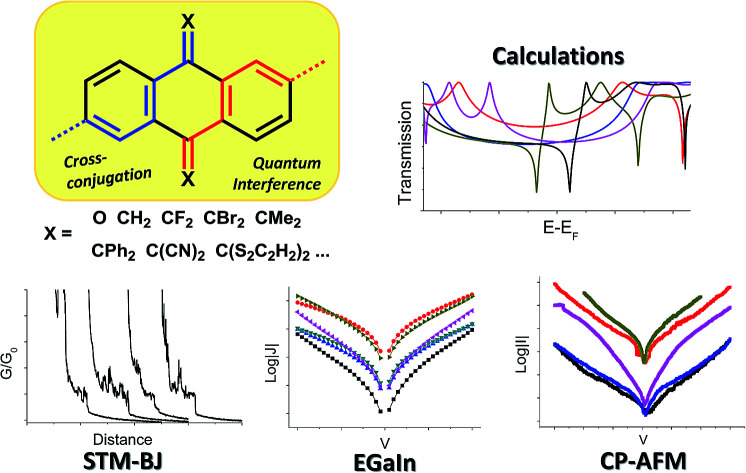

## Introduction

The field of molecular electronics aims to investigate the charge transport through single molecules and molecular ensembles with the goal of translating their electronic and steric properties into functional devices that can interface with modern integrated circuits. In this sense, molecules are particularly interesting thanks to their intrinsic quantum mechanical nature that can give rise to unique phenomena with no straightforward analogs in semiconductor electronics.^1,2^ One of the more well-studied examples of such behaviors is quantum interference (QI), which is a collection of phenomena related to fermions the wave functions of which can interfere with themselves.^3–5^ In the case of molecular tunneling junctions, destructive QI can lower the transmission probability across the molecule, which lowers the conductance between the electrodes by orders of magnitude without altering the tunneling distance, paving the way for hyper-resistive molecular insulators and thermoelectric materials.^6,7^ Systems in which QI can be toggled on and off by external stimuli are of paramount interest to design devices that can scale molecular inputs to macroscopic outputs, such as molecular switches, memories, and transistors.^8^ For these reasons, the effects of QI on molecular charge transport have been the subject of both theoretical and experimental studies across multiple platforms.

As for π-conjugated molecules, QI effects are often observed in molecules with cross-conjugation,^9–12^*meta*-substitution,^13,14^ or peculiar spatial arrangements.^15,16^ To be able to translate these functionalities in actual devices, it is necessary not only to understand the relations between QI and charge transport on a fundamental level but also to translate this knowledge to design principles that can be used to achieve a better structure–function control in device-relevant contexts. The realization of the latter is still largely lacking. One reason is that phenomenological observations are often reported for a specific molecule in a specific experimental context and platform to elucidate a fundamental relationship or property rather than a function. To exploit QI phenomena as functional control over conductance, the energy of the interference feature has to be close to the Fermi level (*E*_f_) inside the assembled junction. The most basic level of control is using functional groups with different electron-withdrawing/donating properties to affect the electronic levels of the molecule and move the energy of the feature, but without the guidance of empirical relationships, the position of the feature can be far from optimal.^14,17–19^

Molecules containing an anthraquinone core are often cited as examples when discussing the relation between cross-conjugation and QI in tunneling junctions,^11,20–24^ but the effect of the conjugation pattern itself and the role of the electronegativity of the oxygen atoms in such structures have only been marginally addressed in the literature.^25^ Molecular wire-like compounds incorporating anthraquinone present many advantages such as chemical inertness, straightforward preparation and well-characterized redox properties. Yet, in a scenario where the electronic properties of the wires are of paramount importance for the investigation of QI effects in a junction, the quinoid functionality offers comparatively little tunability as a cross-conjugated moiety. Nonetheless, it is a readily accessible synthon for systematic investigations into QI in π-conjugated molecules.

**Fig. 1:**
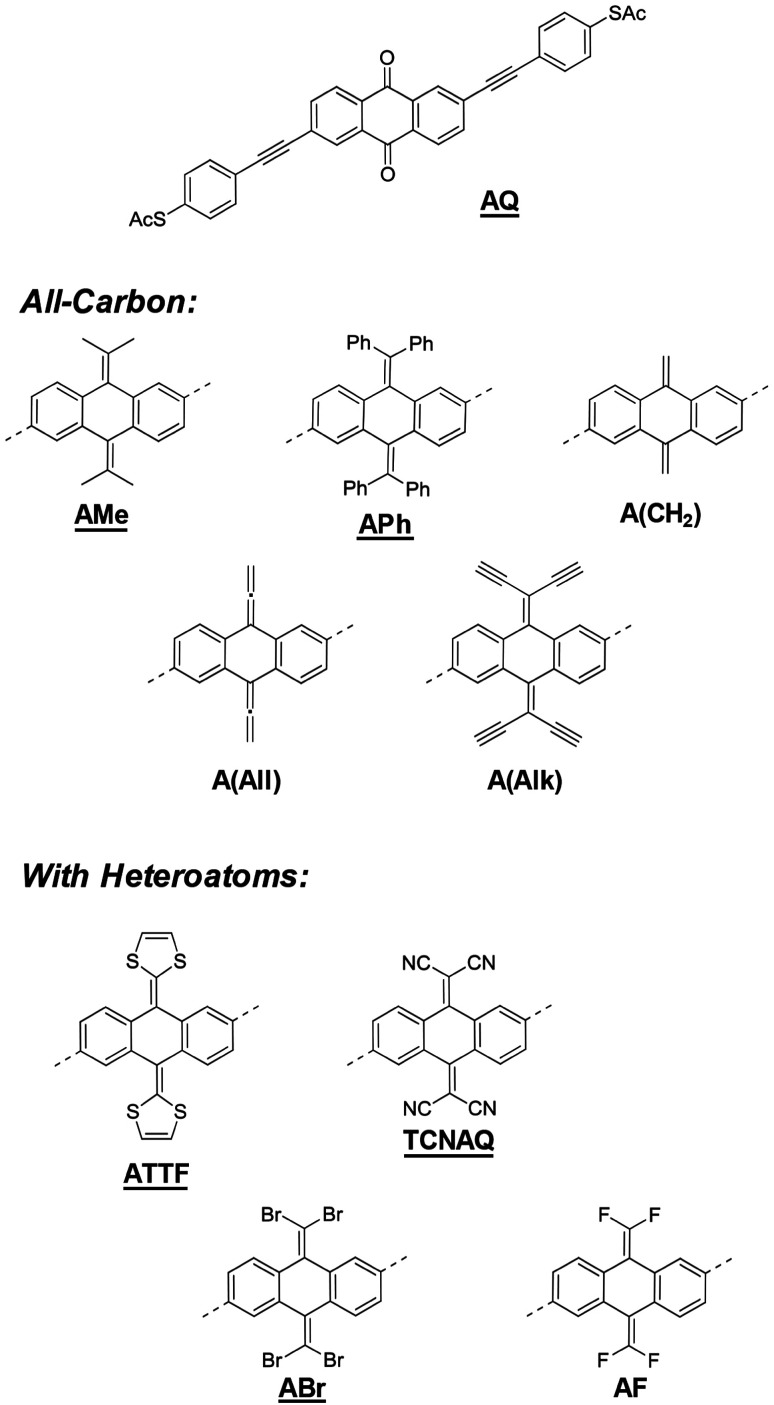
The molecules investigated in this study. The underlined compounds were successfully synthesized and isolated. The cores of the all-carbon and heteroatom-containing molecules are attached to the same phenylacetylene arms as AQ at the positions indicated with dashed lines.

In this work, we discussed a series of molecular wires with identical molecular skeletons and binding groups to the parent anthraquinone wire (AQ),^11^ but with different CX_2_ groups in place of the oxygen in the carbonyl, thus changing energies and localization of the molecular orbitals (MO), molecular geometry, and the distribution of electron density, without altering the cross-conjugated core. The compounds are shown in Fig. 1. We investigated their tunneling charge-transport properties both *in silico* and in two different experimental platforms, namely, single-molecule scanning tunneling microscope break-junctions (STM-BJs) and large-area junctions comprising self-assembled monolayers (SAMs) using conducting probe-AFM (CP-AFM) and liquid eutectic Ga–In (EGaIn) top contacts.^26^ We also discuss the accessibility of molecular modifications synthetically, including some that have been studied theoretically but are unstable or otherwise experimentally inaccessible.

With this approach we were able to (I) gain a better understanding of the nature of carbonyls as cross-conjugating units; (II) investigate, both theoretically and experimentally, the effect of side CX_2_ groups on the position of the QI resonance with respect to AQ; (III) observe how the degree of orbital overlap *vis-à-vis* the torsion angle of the cross-conjugated core (caused by steric bulk) affects QI; (IV) explore the parameter space of chemical modifications that are accessible starting from the anthraquinone core of the wires and their stability; and (V) isolate many platform-specific variables such as electrode composition and collective effects.

## Design and transport calculations

Fig. 1 shows a series of molecular wires characterized by an anthraquinoid core but bearing different functionalities at the 9 and 10 positions. *Via* a carbon–carbon double bond in place of the carbonyl group, it is possible to investigate the effect of the quinone moiety on tunneling transport compared to compounds that have the same conjugation pattern but are not quinones (*i.e.*, they are quinoids).

Our focus is on compounds that have been proposed and studied theoretically but are yet to be synthesized and studied experimentally, such as A(CH_2_) and TCNAQ.^20^ Others have been reported but were investigated narrowly for very specific phenomena like the redox properties of ATTF.^27^ We also include in the series molecules that are structurally/electronically similar, for example substituting Br for CN, to investigate the influence of small variations in the molecular identity on tunneling charge transport. The presence of exocyclic sp^2^ carbons can allow the manipulation of the electronic properties of the molecule synthetically over a wide range of functionalities. The insertion of side groups with different electron-donating/withdrawing and steric properties can affect both the energy landscape of the molecule and the geometry of the core. The effect of the various substitutions on the frontier orbitals of the molecules, which dominate tunneling charge transport, together with the bent-angle of the anthraquinoid core (*ϕ*, see ESI†) is summarized in Table 1. The synthesis of some of the compounds in Fig. 1 may have been attempted but, as we discovered, was impossible or led to unstable products, which we believe is important to report, as we do in this work.

**Table 1:** Calculated HOMO, LUMO and frontier orbital gaps and angles for the wires proposed in Fig. 1^a^

	AQ	AMe	APh	A(CH_2_)	A(All)	A(Alk)	ABr	ATTF	TCNAQ	AF
LUMO (eV)	−3.24	−1.72	−1.87	−2.05	−1.92	−2.79	−2.35	−1.91	−3.99	−2.21
HOMO (eV)	−5.98	−5.44	−5.48	−5.62	−5.44	−5.60	−5.80	−4.86	−6.19	−5.77
Band gap (eV)	2.74	3.72	3.61	3.56	3.52	2.80	3.45	2.94	2.20	3.56
*ϕ* (degree, °)	0	47	47 (45)	27	0	38	47 (44)	36	36	31

a Numbers in parentheses are from X-ray crystal structures.

The energies of the frontier orbitals for the all-carbon derivatives A(CH_2_), AMe, APh, and A(All) are similar [(−5.5 ± 0.1) eV and (−1.9 ± 0.2) eV, respectively], showing that the effect of these substituents on the electronics of the core is comparable; however, there are clear differences between these compounds and AQ, the LUMO and HOMO of which are more than 1 eV and 0.5 eV lower in energy, respectively. The smaller frontier orbital gap (*E*_g_) for A(CH_2_) and A(All) compared to AMe and APh correlates with the degree of planarity of the molecule, *i.e.*, a higher degree of conjugation (see ESI†). Interestingly, A(Alk) is an outlier in the hydrocarbon series: its LUMO is localized on the core, does not span the whole molecule between the electrodes (Fig. S18†) and has a relatively low energy (only 0.5 eV above that of AQ), demonstrating the surprising electron-withdrawing properties of the ethynyl moieties.

When heteroatoms are introduced, the effects on the orbital energies are more dramatic, which is particularly obvious for compounds bearing extended tetrathiafulvalene^27^ or tetracyanoanthraquinodimethane cores (ATTF and TCNAQ, respectively). These cores are known for their redox properties, which make the former a good electron donor and the latter a good electron acceptor. Indeed, the desirable properties of these cores are preserved in the wires: ATTF is characterized by the highest-lying HOMO in the series (1.1 eV higher than that of AQ), while TCNAQ exhibits the lowest-lying LUMO (0.8 eV lower than that of AQ). The functionalization of the anthraquinodimethane core with four halogen substituents, as in the cases of ABr and AF, lowers the energy of both frontier orbitals but not as effectively as the aforementioned compounds.

Using a simple, 2D tight-binding model, all the cross-conjugated compounds are predicted to show destructive QI as a result of the bond alternation (Fig. S16†).^5,20,28^ However, the presence of pendant groups bearing different substituents changes the electronics of the molecule and can affect the position and the shape of the QI feature with respect to the Fermi energy (*E*_f_) inside the junction, thereby affecting the measured conductance differently. This phenomenon has been explored theoretically and experimentally in the case of meta-substituted benzene and biphenyl in single-molecule junctions, but not in large-area junctions.^14,18,19,29^ Moreover, the torsional angle introduced in the core by the bulky pendant groups might also affect the QI features by means of through-space interactions, which are expected to differ in single-molecule junctions and in monolayers, in which molecules tend to planarize.^16^

**Fig. 2:**
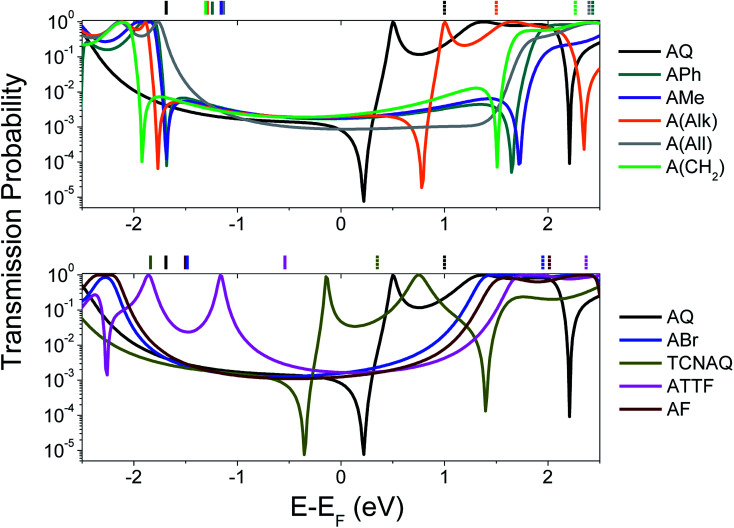
Calculated transmission probability as a function of electron energy (with respect to *E*_f_ = −4.3 eV; see ESI† for more details) of different molecular wires. Top: all-carbon wires; bottom: wires containing heteroatoms. AQ is reported in both the plots as a reference. The gas-phase DFT HOMO (solid lines) and LUMO (dashed lines) energies are shown across the top *X*-axis with respect to the *E*_f_. The non-reported LUMO values were characterized by *E*_LUMO_ − *E*_f_ > 2.5 eV.

To gain a better understanding of the overall effect of the substituents on the charge transport across the molecule, we computed the transmission probability as a function of energy for various molecules as shown in Fig. 1. The results are shown in Fig. 2.

The calculations were performed on single molecules, as calculations on SAMs of these molecules are prohibitively complex and the details of molecular conformation and packing are unknowable. Although the properties of molecules are affected by collective effects that may arise in a SAM (*e.g.*, the alignment of dipole moments and effects on the geometry of the molecules),^8,16,30,31^ studies showed that it is possible to achieve good qualitative agreements between single-molecule calculations and experimental results in large-area molecular junctions comprising SAMs,^25,32^ particularly with trends.

The spectra shown in Fig. 2 feature sharp dips in transmission, which we attribute to destructive QI. When the energy of the dip is found near the *E*_f_, QI can substantially depress the tunneling probability between the two electrodes, which we observe as a decrease in conductance.

As can be seen in the top panel of Fig. 2, the all-carbon wires (except A(All)and A(Alk)) show analogous transport properties, comparable to what was already predicted for A(CH_2_) by Valkenier *et al.*^20^A(All), on the other hand, only shows a suppressed transmission, meaning that the position of the interference dip with respect to the *E*_f_ is significantly different from that of the former compound. There are several potential causes for this difference; the angle of the core is remarkably different, with A(All) (just like AQ) being the only molecule that is completely planar (see Table 1). However, altering the torsional angles (*φ*) *in silico* only shifts the energy of the feature by a few meV without affecting the shape or general features (Fig. S20 and S21†).^33^ That steric effects only affect the charge transport marginally when compared to electronic contributions is also evident from the comparison of the spectra in Fig. 2 and *φ* in Table 1, for which there is no obvious trend. More likely is that the difference we observed has to do with the different hybridization of the central carbon of the allene compared to the methylene of A(CH_2_) (*i.e.*, sp instead of sp^2^). The allene moiety, despite being traditionally seen as having the carbons at positions 1 and 3 as non-conjugated, in not innocent and does not behave like a simple carbon–carbon double bond in the case of tunneling transport,^34^ thus making the case of A(All) significantly different from that of A(CH_2_). This is also noticeable in the different spatial distribution and symmetry of the highest occupied π-state (HOPS) and the lowest unoccupied π-state (LUPS) of these two molecules (Fig. S18†). The transmission probability plots of ABr and AF are also featureless and similar in magnitude to those of the former all-carbon derivatives around the *E*_f_. Apparently, the similarity in the electronic characteristics of these two wires is reflected in their transport properties, irrespective of the differences in the electronegativities of the halogen atoms and the fact that *ϕ* for AF is smaller. Likewise, ATTF does not show any QI feature in the frontier orbital gap. Yet, its line-shape in Fig. 2 differs considerably from that of the compounds discussed thus far. The high-lying HOMO (the highest in the series) results in a high-transmission resonance 0.9 eV below the *E*_f_.

Interestingly, the three molecules characterized by the lowest LUMO energies in the entire series—A(Alk), AQ, and TCNAQ—show a single, sharp feature. Starting from the former, the feature falls closer to the *E*_f_ as the LUMO energy decreases; as the electron-withdrawing character of the pendant group increases (*i.e.*, the LUMO moves down in energy), the energy of the interference feature is shifted to lower values.^18^ It was an interesting correlation that AQ, TCNAQ, and A(Alk) with their transmission spectra shifted towards lower electron energies have a core-localized LUPS, while ATTF that has a transmission spectrum shifted towards higher electron energies has a core-localized HOPS, a correlation that we previously observed in benzodithiophenes.^25^

In this section, we discussed how different molecular properties can affect the transmission between the electrodes using computational methods. We argue that one of the limitations of using AQ to investigate the correlations between QI and cross-conjugation is its peculiar energetic situation: if a low lying and/or core-localized LUMO is necessary to observe the QI feature in anthraquinoid molecules, then there are only a few compounds we can access bearing functionalities with a comparable (or increased) electron-withdrawing behavior with respect to AQ. While an *in silico* approach allows us to vary the molecular energetics continuously and at will, in static molecular junctions (*e.g.*, devices) we can only observe the properties of synthetically accessible compounds in a particular conformation.

## Synthesis

**Fig. 3:**
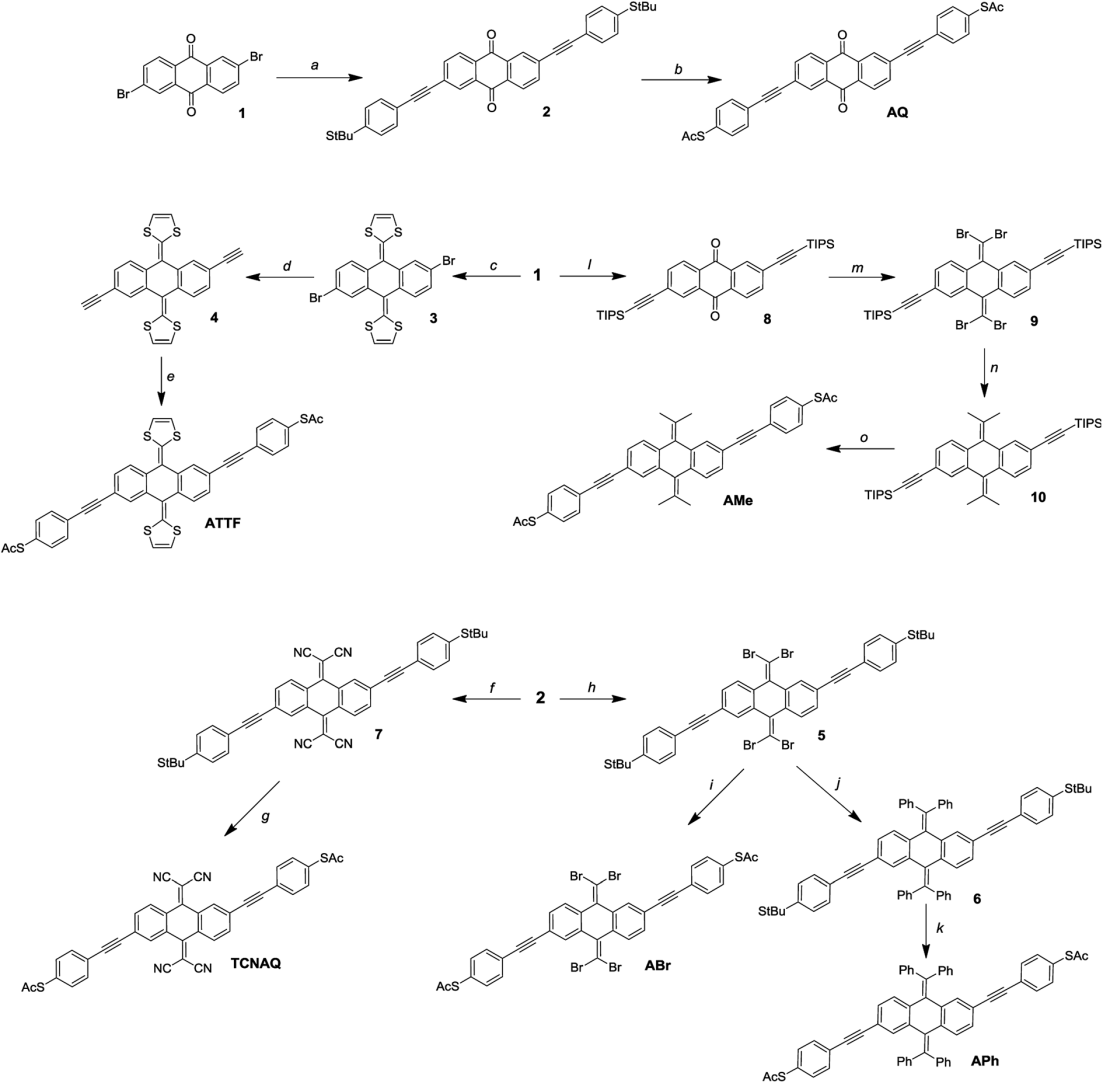
Reaction scheme for the synthesis of the wires: (a) *tert*-butyl(4-ethynylphenyl)sulfide, Pd(PPh_3_)_4_, CuI, NEt_3_, THF; (b) BBr_3_, AcCl, DCM/toluene 1 : 1; (c) dimethyl-(1,3-dithiol)-2-ylphosphonate, *n*BuLi, THF; (d) (1) TMS-acetylene, Pd(PPh_3_)_4_, CuI, NEt_3_; (2) TBAF, H_2_O, THF; (e) *S*-(4-iodophenyl)ethanethioate, Pd(PPh_3_)_4_, CuI, NEt_3_, THF; (f) malononitrile, TiCl_4_, pyridine, CHCl_3_; (g) AcCl, TiCl_4_, DCM; (h) PPh_3_, CBr_4_, DCM; (i) BBr_3_, AcCl, DCM/toluene 1 : 1; (j) PhB(OH)_2_, K_2_CO_3_, Pd(PPh_3_)_4_, toluene; (k) BBr_3_, AcCl, DCM/toluene 1 : 1; (l) TIPS-acetylene, Pd(PPh_3_)_4_, CuI, NEt_3_, THF; (m) PPh_3_, CBr_4_, DCM; (n) *B*-MeO-9-borabycyclononane, MeLi, Pd(PPh_3_)_4_, THF; (o) (1) TBAF, H_2_O, THF; (2) *S*-(4-iodophenyl)ethanethioate, Pd(PPh_3_)_4_, CuI, NEt_3_, THF.

It is not uncommon in molecular electronics to find theoretical studies predicting interesting properties in compounds without regard to synthetic accessibility.^20,35,36^ Practical synthetic constraints often require extrapolating these findings to analogous compounds to validate them experimentally. These constraints, in turn, inform theorists which compounds and structural motifs warrant further investigation. As shown in Fig. 3, the synthesis of AQ from the 2,6-dibromo-anthraquinone (1) core is straightforward according to literature procedures.^11^ An ideal convergent synthesis would begin by derivatizing 1 and follow the same route as AQ; however, the stability of 1 and the functional group tolerance of Sonogashira chemistry precluded that approach.

Of the proposed compounds in Fig. 1, ATTF was the most straightforward to synthesize from 1: first, we obtained 3 from dimethyl 2-(1,3-dithiole)phosphonate, and then we prepared 4 (the 2,6-diethynyl derivative of 3) *via* Sonogashira coupling. Following desilanization, we prepared the final wire using Pd/CuI mediated cross-coupling between 4 and (4-iodo)phenylthioacetate. The other compounds in Fig. 1 required the installation of functional groups later in the synthetic route: in the case of TCNAQ, for instance, the malononitrile derivative of 1 cannot be cross-coupled using Sonogashira chemistry.^37^ Thus, we installed the functional group on 2 to form 7, from which we obtained the dithioacetate-wire with one more step.

Following the same approach, we prepared ABr using the Corey–Fuchs reaction to obtain 5, followed by the replacement of the *tert*-butyl groups. This dibromovinyl functionality is also a useful synthon, allowing the preparation of the all-carbon derivatives APh and AMe*via* Suzuki coupling. It is worth mentioning that, compared to the other compounds, the latter tends to become dark within days if not kept in the dark, which is an indication of the relative instability of many anthraquinoids towards Gilch-like polymerization.^38^

While we were able to synthesize these aforementioned compounds, the others presented in Fig. 1 remain elusive. All attempts to prepare A(CH_2_), AF, A(All), and A(Alk) were either unsuccessful or the final compounds could not be isolated, presumably because they lack sufficient steric bulk to inhibit spontaneous polymerization. A(CH_2_), for example, is effectively two styrene molecules locked in-plane and thus very prone to polymerization. These findings are discussed in detail in the ESI.†

Unfortunately, many anthraquinoid molecules are not accessible by chemical synthesis and therefore cannot be studied in any experimental platform. Often the compounds can be prepared but either cannot be isolated or decompose too quickly to perform conductance measurements, which is a fundamental limitation that cannot be circumvented. While we could not prepare compounds such as A(CH_2_) and AF, we were still able to prepare others that show similar transport properties when compared to these former *in silico*, as it is the case for APh and ABr.

### Preparation and characterization of self-assembled monolayers

Single-molecule junctions are useful for validating theoretical and computational models because they can be described accurately *in silico*, but static single-molecule devices have thus far presented an insurmountable challenge. Ensemble junctions, by contrast, are prohibitively complex to model accurately but can be translated to devices.^8,39^ The dynamic nature of SAMs and the collective effects present in molecular ensembles make them particularly difficult to study and to elaborate *in silico*.^8,16,30,31^

We grew SAMs of the compounds mentioned in the previous section on Au-on-mica (Au^Mica^) and template-stripped Au (Au^TS^).^40^ These Au substrates are both atomically flat but different in nature, the former being characterized by highly crystalline Au(111) terraces and the latter by mostly flat amorphous grains of Au (Fig. S8 and S9† respectively). Care has to be taken when growing SAMs of bis-functionalized molecules (such as the ones presented here) as they could tend to lay flat on the surface.^41^ The nature and the quality of the SAMs on Au^Mica^ were investigated using standard and synchrotron photoelectron spectroscopy (see ESI†). For the compounds introduced earlier, with the exception of AMe, the molecules were found to stand upright with only one sulfur bound to the gold, which can be determined from the XPS spectra of the S 2p region. Most of the wires produced two signals: one at ∼164.0 eV compatible with the S atoms on the top of the SAM and another at ∼162.0 eV that we ascribed to Au-bound sulfur. The analyzed SAMs showed a similar packing density and tilt angle with respect to the metal surface (see ESI†). The reasons why AMe does not form good SAMs are unclear but could be related to the reactivity of the molecule: the difficulties encountered during the synthesis as well as the limited air-stability of AMe show that the compound is more reactive than the other wires and, in the closely packed environment of the SAM, could indeed show further reactivity that inhibits the formation of a densely packed SAM. Among the proposed series, the SAM of TCNAQ showed a peculiar feature: the XPS signal of the N 1s orbital was characterized by an extra peak that is not observed for the unbound molecules, which we ascribed to reduced nitrogen species. The characterization of this SAM is reported in depth elsewhere.^42^

Physisorbed monolayers of tetracyanoquinodimethane (which constitute the core of our wire) and related molecules on Au and other noble metals are known to generate spontaneous charge transfer from the metal to the molecules, the reduced state of which can be observed in the XPS signal.^43^ In the case of SAMs of TCNAQ, it is worth mentioning that the presence of even a small fraction of reduced molecules could have drastic effects on the charge transport properties of the monolayer by forming a linearly conjugated and more conductive species.^42,44^

Cases like those of AMe and TCNAQ shed light on another issue that can limit the connection between theory and the preparation of working devices, namely, the translation of molecular properties across different environments. While one can address the physical and chemical characteristics of a molecule using both computational and experimental techniques—from the isolated gas-phase scenario to the infinite close-packed crystal—it is still hard to predict the behavior of an ensemble of molecules in a 2D matrix bound to a metal. A better understanding of the latter is necessary in order to move closer to the realization of real devices: specific functionalities that are chosen by design to generate certain effects may behave in unpredictable ways in the complex environment of a device in virtue of their specific chemical nature, thus complicating the role of certain compounds as materials in molecular electronics.

### Electrical characterization of molecular junctions comprising self-assembled monolayers

Molecular tunneling junctions comprising SAMs are of primary technological relevance for the realization of molecular-electronic devices.^1^ When compared to single-molecule junctions, the former are more complex to model and it is not clear what functionalities can be translated efficiently to large-area devices.^45^ For this reason, an empirical approach can be useful in addressing the electrical properties of SAMs, and it is particularly effective when they are investigated and compared across different platforms and substrates.

**Fig. 4:**
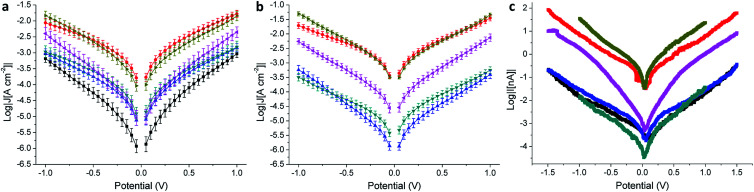
Electrical characterization of tunneling junctions obtained as Au^Mica^/SAM//EGaIn (a), Au^TS^/SAM//EGaIn (b), and Au^Mica^/SAM/Au^CP-AFM^ (c) comprising AQ (black), AC (red), APh (cyan), ABr (blue), ATTF (pink), and TCNAQ (yellow). Error bars in (a) and (b) are confidence intervals (*α* = 0.05). Error bars in (c) are omitted for clarity.

We evaluated the electrical properties of the SAMs in large-area Au^Mica^ or Au^TS^/SAM//EGaIn junctions and small-area Au^Mica^/SAM//Au^CP-AFM^ junctions using CP-AFM (where ‘/’ and ‘//’ denote covalent and van der Waals interactions, respectively). All EGaIn junctions were measured in a nitrogen atmosphere containing 1% to 3% O_2_ and relative humidity below 15%, the details of which are described elsewhere.^46^ We believe that the use of different experimental techniques is of paramount importance to separate the intrinsic molecular properties from features arising from the method employed. Next to the cross-conjugated wires presented in Fig. 1, we also measured as a reference a wire of similar length that is linearly conjugated, bearing an anthracene core substituted at the 2 and 6 positions by the same (ethynyl)phenyl-thioacetate arms (AC, the structure of which is shown in the ESI†). EGaIn is a liquid metal at room temperature that forms soft, non-damaging, conformal contacts with SAMs. Due to a sub-nm layer of Ga_2_O_3_, it exhibits non-Newtonian properties^47^ that enable it to be shaped easily into sharp tips (with a diameter of about 20 μm), which are used to form the junctions in a fast and reproducible manner. Neither the sample nor the EGaIn electrode is destroyed during the measurement, and therefore this methodology enables the formation of junctions in multiple areas of a substrate, allowing the collection of statistically significant data.^26^ The results for the EGaIn measurements are shown in Fig. 4a and b and summarized in Table 2. The dataset we used for AQ on Au^Mica^ was already reported in a previous study.^25^

**Table 2:** Summary of electrical characteristics of large area Au/SAM//EGaIn junctions

		AQ	AC	APh	ABr	ATTF	TCNAQ
Au^Mica^	Yield (%)	96	77	56	85	76	69
	log|*J*| (0.1 V)	−5.5 ± 0.2	−3.4 ± 0.1	−4.5 ± 0.2	−4.7 ± 0.2	−4.6 ± 0.2	−3.7 ± 0.2
	log|*J*| (0.75 V)	−3.5 ± 0.1	−2.1 ± 0.2	−3.2 ± 0.2	−3.2 ± 0.1	−2.9 ± 0.2	−2.3 ± 0.2
Au^TS^	Yield (%)	—	98	100	100	100	100
	log|*J*| (0.1 V)	—	−3.1 ± 0.1	−5.0 ± 0.1	−5.5 ± 0.2	−4.2 ± 0.2	−3.2 ± 0.1
	log|*J*| (0.75 V)	—	−1.8 ± 0.1	−3.7 ± 0.1	−4.0 ± 0.1	−2.6 ± 0.1	−1.8 ± 0.1

As can be seen in the *J*/*V* plot for Au^Mica^/SAM//Ga_2_O_3_/EGaIn junctions (Fig. 4a), through most of the bias window, the current densities of the investigated SAMs follow the order TCNAQ ≈ AC > APh ≈ ABr ≈ ATTF > AQ. This result suggests no dependence of the conductance on the torsional angle of the core as predicted by our calculations; *i.e.*, the degree of conjugation between the ends of the wire does not strongly affect the tunneling probability. Surprisingly, TCNAQ is as conductive as AC, despite the latter being linearly conjugated; however, this unusually high conductance can be ascribed to the partial reduction of SAMs of TCNAQ, which, in contrast to the neutral molecule, is linearly conjugated: although only a small fraction of the molecules in the SAM is found in that state, by drawing an analogy with the current that flows through a series of resistors in parallel, 1–2% of more conductive molecules are enough to dominate the charge transport across the whole junction.^48^ The unusual properties of tunneling junctions comprising TCNAQ SAMs are described in detail elsewhere.^42^

The shapes of the *J*/*V* curves of ATTF and AQ are steeper and more symmetrical than those of the other molecules, which causes ATTF to be more conductive than APh and ABr at higher biases. A slight asymmetry in metal/SAM//EGaIn junctions comprising conjugated molecules is expected because of the non-identical interfaces between the two ends of the molecules and the electrodes. In the case of AQ, we ascribed the *J*/*V* characteristics of the junctions to QI. We discussed this observation in detail in a previous study where we showed that the differential conductance plot (log|d*J*/d*V*|) of Au^Mica^/AQ//Ga_2_O_3_/EGaIn junctions is characterized by negative curvature.^25^

**Fig. 5:**
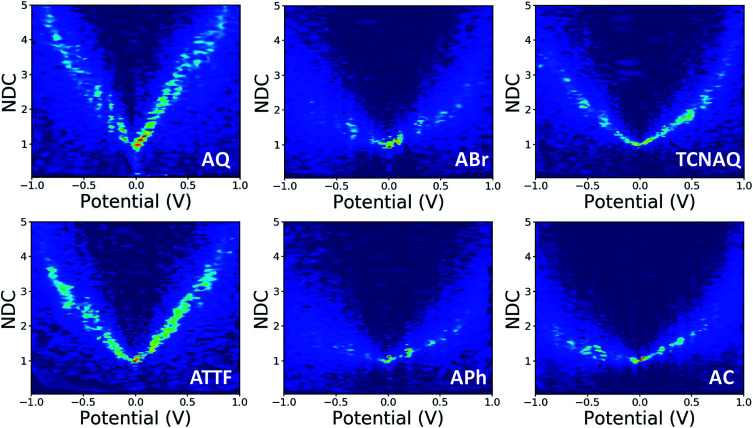
Normalized differential conductance heatmaps for Au^Mica^/SAM//EGaIn junctions comprising AQ, ABr, TCNAQ, ATTF, APh, and AC.

In this study, we wanted to better highlight the differences in the *J*/*V* line shapes for the different systems and gain better insight into the transport mechanism. For these reasons we decided to plot the collected data as normalized differential conductance (NDC).^49^ Compared to the plots discussed above, in NDC heatmaps the systems are represented on the same scale independently of the magnitude of the current density and can be easily compared, revealing information about transmission features close to the *E*_f_.^50^ The results are shown in Fig. 5. The NDC heatmaps for AQ and ATTF are significantly steeper and sharper around 0 V compared to those for the other compounds. While for AQ we ascribed this finding to QI, we believe the explanation to be different in the case of ATTF: for this system, we ascribe such a line shape to the low lying HOMO of this molecule, which can also result in a rapidly increasing tunneling probability around the *E*_f_ (Fig. 2) which is also responsible for the higher |*J*| at higher bias when compared to APh and ABr.

The plot of TCNAQ is also interesting as it describes a bowl shaped NDC though the QI feature is predicted to be less than 0.2 eV away from the *E*_f_. This could result from the presence of (linearly conjugated) reduced molecules in the SAM which dominates the conductance characteristics of the junction.^42^

In the case of Au^TS^/SAM//EGaIn junctions (Fig. 4b and Table 2), we found a similar trend in *J* to Au^Mica^ (Fig. 4a) with some differences: (I) the current density values for the different SAMs are spread over a larger codomain of log|*J*|; (II) the yield of the working junctions was significantly higher; (III) the statistical variance in *J* was smaller; and (IV) it was not possible to measure AQ because of the extremely low yield of the working junctions (specifically under the low O_2_/humidity conditions used throughout this study). These results show that, although the nature of the SAM (and hence the data collection) is influenced by the substrate, the transport characteristics of the identical molecules on the different Au surfaces are comparable and show similar properties. Compared to the case of Au^Mica^, ATTF appears now to be more conductive than APh and ABr over the entirety of the accessible bias window; this observation could be due to the wider distribution of the values of *J* in the case of Au^TS^ which can enhance the differences between the different compounds (Fig. 4b).

We observed the same trend in Au^Mica^/SAM//Au^CP-AFM^ junctions (Fig. 4c and Table 3), in which again the conductance of ATTF is significantly different from that of the other compounds. The trend in log|*J*| for the different SAMs was maintained with the exception that we were not able to distinguish between AQ, APh, and ABr, because of the low currents produced by the smaller contact area of this technique (Fig. S13†). Compared to EGaIn junctions, by using an AFM tip as the top electrode we can contact an area several orders of magnitude smaller (70–100 molecules):^51^ the fact that the trend is preserved across the series excludes the influence of defects and/or artifacts that are present in large-area junctions as determining factors in conductance of the junctions. This is of particular relevance in the case of TCNAQ, indicating that the high conductance is indeed a property of the SAM on Au and not an observation only relative to the Au/SAM//EGaIn system. It is also important for the potential application of large-area tunneling junctions in devices.

**Table 3:** Summary of electrical characteristics of small area Au^Mica^/SAM//Au^CP-AFM^ junctions

	AQ	AC	APh	ABr	ATTF	TCNAQ
log|*J*| (0.1 V)	−3.6 ± 0.2	−1.3 ± 0.1	−3.9 ± 0.4	−3.4 ± 0.2	−2.9 ± 0.2	−0.8 ± 0.4
log|*J*| (0.75 V)	−2.1 ± 0.2	0.3 ± 0.3	−2.0 ± 0.3	−2.0 ± 0.2	−0.5 ± 0.2	0.9 ± 0.4

In this section we investigated the electrical properties of SAMs of anthraquinoid compounds on two different experimental platforms and found that their conductances roughly follow the order TCNAQ ≈ AC > ATTF > APh ≈ ABr > AQ. These findings show that the large-area techniques used are sensitive enough to differentiate molecular junctions comprising molecules with identical bond topology and different electronic structures but only when the latter change drastically (as it is expected from the calculation) or the nature of the molecule is not changed by the interaction with the substrate. When new functionalities are incorporated into a molecule to affect the transmission probability, they influence steric and electronic properties as well, which may contribute to the overall properties of the junctions and the SAMs in general. Surprisingly, steric effects play a negligible role in tunneling charge-transport through SAMs.

### Single molecule junctions

**Fig. 6:**
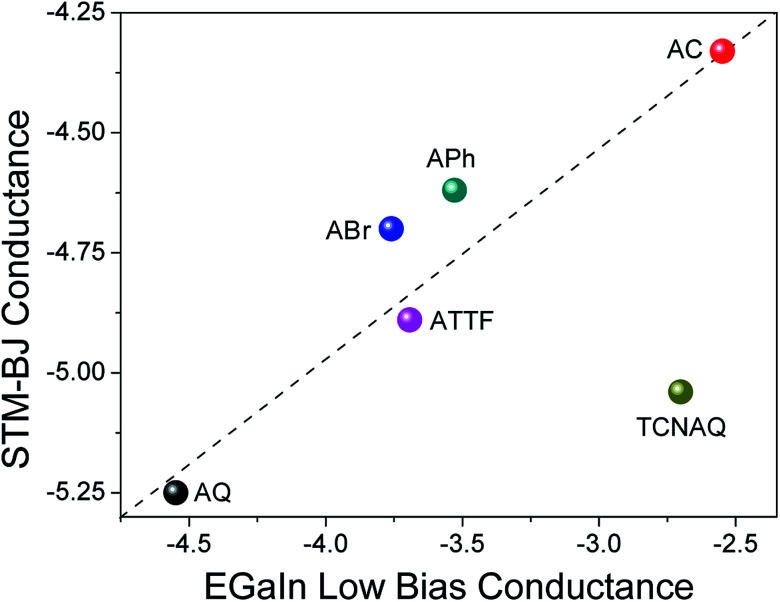
Confrontation between the values of conductance obtained for single-molecule STM-BJ at 0.1 V (*y*-axis) and low-bias conductance extracted from Au^Mica^/SAM//EGaIn junctions (*x*-axis). Both data sets are presented on a logarithmic scale. The dashed line is intended as a guide to the eye for deviation from linearity.

To gain further understanding of the transport properties of the proposed molecules opposed to effects that may arise in the SAM, we measured the compounds in single-molecule junctions using an STM-BJ setup (Fig. 6). We found surprisingly good (nearly linear) agreement between the two data sets: the conductance of AC was the largest in both STM-BJ and EGaIn junctions, and that of AQ was the lowest, while ABr, APh, and ATTF fell somewhere in the middle. This trend was expected from the theoretical transport calculation (Fig. S19†), where AQ and TCNAQ were the only two compounds of the synthesized series to show a pronounced QI dip near the *E*_f_. In tunneling junctions comprising SAMs, the behavior of TCNAQ deviated sharply from theory; however, in single-molecule junctions its conductance was reduced and similar to that of AQ. This observation highlights the unexpected role the SAM can play in determining the electrical properties of a large-area junction. The broadening that one might expect from intermolecular interactions appears to have little effect on tunneling charge-transport; rather, it is the stabilization of charges in ensembles of TCNAQ that makes the difference.^42^

Altogether, these observations show that, for the most part, the experimental trends in conductance in single-molecule and ensemble junctions using different electrodes, substrates and contact areas are preserved and can relate to theoretical calculations. This is remarkable if we consider the different variables that affect the different measurements and are an intrinsic part of the different methodologies used. However, cross-platform characterization remains the best method for establishing structure–function relationships because it ensures that a particular observation is not tied to a unique experimental platform and is likely to be preserved in whatever device platforms emerge in the future.^8^

## Conclusions

The aim of this study was to investigate the charge transport properties of a series of cross-conjugated molecular wires characterized by a molecular skeleton identical to AQ but with different electronic structures and torsional angles. We achieved this both *in silico* and across different experimental platforms, including both single-molecule and large-area junctions. We found similar trends between the calculated transmission probability and the different experimental platforms despite the myriad factors external to the gas-phase electronic and physical structure of molecules that can lead to very different properties in different experimental contexts. Thanks to this approach we were able to find strong evidence that ultimately the level-alignment in assembled junctions will determine the observed conductance and that the torsional angle of the core seems to affect QI to a surprisingly small extent in the case of anthraquinoid compounds in contrast to the observation in other systems,^16,52^ thus making the former interesting candidates for potential application in real devices. The analogies we found, however, are limited to the subset of the molecules studied computationally that we were able to synthesize and measure: compounds like A(CH_2_) have been proposed in the literature^20^ but cannot be prepared because the tendency of anthraquinoids to polymerize is exacerbated by electron-withdrawing/donating substituents, requiring substantial steric bulk to produce stable compounds. While the distortions to the π-system imposed by this bulk have a surprisingly small effect on tunneling charge transport, it limits the scope of accessible functional groups. One important finding of this study is that anthraquinone is a terrible platform for tuning QI features synthetically. We suggest benzodithiophenes as an alternative.^25^

## Conflicts of interest

There are no conflict to declare.

## Supplementary Material

NA-001-C8NA00223A-s001
