# Correction: Systematic experimental study of quantum interference effects in anthraquinoid molecular wires

**DOI:** 10.1039/c9na90018g

**Published:** 2019-03-28

**Authors:** Marco Carlotti, Saurabh Soni, Xinkai Qiu, Yong Ai, Eric Sauter, Michael Zharnikov, Ryan C. Chiechi

**Affiliations:** Zernike Institute for Advanced Materials Nijenborgh 4 9747 AG Groningen The Netherlands r.c.chiechi@rug.nl; Stratingh Institute for Chemistry, University of Groningen Nijenborgh 4 9747 AG Groningen The Netherlands; Applied Physical Chemistry, Heidelberg University Im Neuenheier Feld 253 Heidelberg 69120 Germany

## Abstract

Correction for ‘Systematic experimental study of quantum interference effects in anthraquinoid molecular wires’ by Marco Carlotti *et al.*, *Nanoscale Adv.*, 2019, DOI: 10.1039/c8na00223a.

The authors regret the omission of one of the authors, Yong Ai, from the original manuscript. The corrected list of authors and affiliations for this paper is as shown above.

The Royal Society of Chemistry apologises for these errors and any consequent inconvenience to authors and readers.

## Supplementary Material

