# Supplementary material for: Systematic experimental study of quantum interference effects in anthraquinoid molecular wires
Source: Nanoscale Adv. 2019 Feb 7;1(5):2018–28. doi: 10.1039/c8na00223a (PMC6592160; doi:10.1039/c8na00223a)
Supplement: NA-001-C8NA00223A-s001 [file NA-001-C8NA00223A-s001.pdf]

Electronic Supplementary Material (ESI) for Nanoscale Advances.
This journal is © The Royal Society of Chemistry 2019

Supporting Information: Systematic Experimental Study of Quantum Interference Effects in Anthraquinoid Molecular Wires

Marco Carlotti,[†,‡] Saurabh Soni,[†,‡] Xinkai Qiu,[†,‡] Eric Sauter,[¶] Michael Zharnikov,[¶] and Ryan C. Chiechi[*,†,‡]

†*Zernike Institute for Advanced Materials, Nijenborgh 4, 9747 AG Groningen, The Netherlands*

‡*Stratingh Institute for Chemistry, University of Groningen, Nijenborgh 4, 9747 AG Groningen, The Netherlands*

¶*Applied Physical Chemistry, Heidelberg University, Im Neuenheier Feld 253, Heidelberg 69120, Germany*

E-mail: r.c.chiechi@rug.nl

1 Materials

All reagents were purchased from Sigma-Aldrich, Acros, or TCI Europe and used as received unless otherwise stated. Triethylamine and $CHCl_3$ were distilled over CaH and P_2O_5 respectively, and used within 10 days. Acetonitrile, dichloromethane (DCM), toluene, tetrahydrofuran (THF) were obtained anhydrous from a house system. NMR spectra were recorded on a Varian AMX400 (400 MHz) and referenced to the solvent peak ($CDCl_3$: H, 7.26

ppm; C, 77 ppm) relative to tetramethylsilane. S,S'-((anthracene-2,6-diylbis(ethyne-2,1-diyl))bis(4,1-phenylene)) diethanethioate (**AC**), 2,6-dibromoanthraquinone (**1**), 2,6-bis((4-(tert-butylthio)phenyl)ethynyl)anthracene-9,10-dione (**2**), S,S'-(((9,10-anthraquinone-2,6-diyl)bis(ethyne-2,1-diyl))bis(4,1-phenylene)) diethanethioate (**AQ**), and S-(4-iodophenyl) ethanethioate (**IPhSAc**, were prepared elsewere.[S1] The procedure for the synthesys of S,S'-(((9,10-bis(dicyanomethylene)-9,10-dihydroanthracene-2,6-diyl)bis(ethyne-2,1-diyl))bis(4,1-phenylene)) diethanethioate (**TCNAQ**) is reported elsewhere.[S2] The structure of **AC** is reported in Figure S1. Template stripped metal substrates were prepared by depositing a 100 nm-thick layer of metal on a Si wafer in a metal evaporator. 1x1x0.3 cm glass slides were glued to the deposited metal using an UV-curable optical adhesive (Norland series 60). The samples were cleaved from the wafer with the help of a razor and immediately used. Au on mica (1x1 cm, 200 nm thick Au) was obtained from Phasys (Switzerland) and kept in the original packing in a glovebox until use.

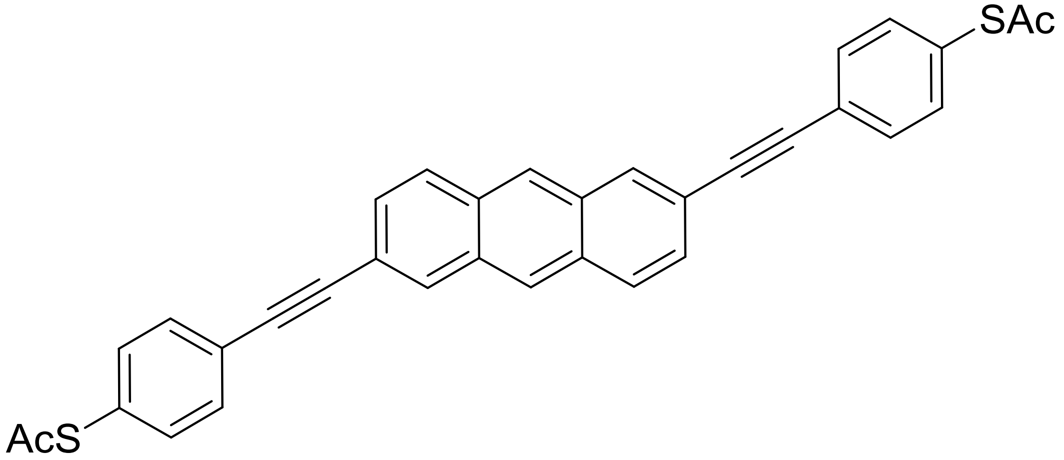

Figure S1: Structure of **AC**.

2 Synthesis

2,2'-(2,6-dibromoanthracene-9,10-diylidene)bis(1,3-dithiole), 3

In a dry 100 mL flask under N_2, 186 mg of dimethyl-(1,3-dithiol)-2-ylphosphonate (0.87 mmol) were dissolved in 50 mL of dry THF and the solution cooled down to −80 °C in a EtOH/dry

ice bath. 0.54 mL of nBuLi 1.6 M in hexanes (0.87 mmol) were added and the solution kept under stirring for 30 minutes. 80 mg of **1** were then added and the reaction left to rise at room temperature overnight. The THF was removed under vacuum. The residue was then dissolved in $CHCl_3$, extracted with water, and dried over Na_2SO_4. The product was finally recrystallized from $CHCl_3$/hexane as red needle-like crystals (80 mg, 68% yield). Multiple batches were prepared with yield 50-70%. ^{1}H-NMR (400 MHz, $CDCl_3$): δ 7.80 (d, J = 1.9 Hz, 2H), 7.54 (d, J = 8.3 Hz, 2H), 7.39 (dd, J = 8.3, 2.0 Hz, 2H), 6.34 (s, 2H). ^{13}C-NMR (101 MHz, $CDCl_3$) δ 140.66, 139.80, 136.71, 131.44, 130.33, 128.97, 122.29, 122.20, 120.00.

((9,10-di(1,3-dithiol-2-ylidene)-9,10-dihydroanthracene-2,6-diyl)bis(ethyne-2,1-diyl))bis(trimethylsilane), 4.1

In a dry Schlenk under N_2, 100 mg of **3** (0.19 mmol) were dissolved in 15 mL of dry THF. 10 mL of freshly distilled NEt_3 were added and the solution bubbled with N_2. 0.14 mL on TMS-acetylene (0.68 mmol) were added, followed by 15 mg of $Pd(PPh_3)_4$ and 5 mg of CuI. The reaction was left at 40 °C for 2 days. The solvents were removed and the reside dissolved in DCM and extracted with HCl 1 M and water until neutrality was reached. The organic phase was then preadsorbed on silica and purified on a short column (SiO_2, hexane/ethylacetate 4:1). A yellow solid was obtained (97 mg, 85% yield). ^{1}H-NMR (400 MHz, $CDCl_3$): δ 7.76 (d, J = 1.5 Hz, 2H), 7.60 (d, J = 8.0 Hz, 2H), 7.36 (dd, J = 8.0, 1.5 Hz, 2H), 6.33 (d, J = 1.3 Hz, 4H), 0.25 (s, 18H).

2,2'-(2,6-diethynylanthracene-9,10-diylidene)bis(1,3-dithiole), 4

In a 250 mL flask, 97 mg of **4.1** (0.17 mmol) were dissolved in 125 mL of THF and the solution was placed in an ice bath. 0.5 mL of tetrabutylammoniumfluoride 1 M in THF (containing 5% vv. water) (0.42 mmol) were added dropwise and the reaction was left stirring at room temperature for 30 minutes. The solution was then filtered through a pad of silica and the solvent removed under vacuum. The product was recrystallized from $CHCl_3$/hexane

as red elongated crystals (50 mg, 68% yield). ^{1}H-NMR (400 MHz, $CDCl_3$): δ 7.79 (d, J = 1.6 Hz, 2H), 7.63 (d, J = 8.0 Hz, 2H), 7.39 (dd, J = 8.0, 1.6 Hz, 2H), 6.31 (s, 4H), 3.10 (s, 2H). ^{13}C-NMR (101 MHz, $CDCl_3$) δ 145.89, 138.26, 137.91, 132.38, 131.01, 127.57, 123.04, 122.17, 120.00, 86.41, 70.61.

S,S'-(((9,10-di(1,3-dithiol-2-ylidene)-9,10-dihydroanthracene-2,6-diyl)bis(ethyne-2,1-diyl))bis(4,1-phenylene)) diethanethioate, ATTF

In a dry Schlenk under N_2, 42 mg of **4** (0.10 mmol) and 90 mg of **IPhSAc** (0.32 mmol) were dissolved in 10 mL of dry THF. 2 mL of freshly distilled NEt_3 followed by 12 mg of $Pd(PPh_3)_4$ and 5 mg of CuI were added and the reaction was left at 40 °C overnight. The solvents were removed under vacuum and the residue dissolved in $CHCl_3$ and water. HCl 1 M was added dropwise until neutrality was reached. The organic phase was then extracted with water, dried over Na_2SO_4, and preadsorbed on silica. The product was purified via column chromatography (SiO_2, hexane:ethylacetate 2:1 increasing to 1:1) and it was recrystallized from DCM/hexane. It was obtained as a red solid (26 mg, 36% yield). ^{1}H-NMR (400 MHz, $CDCl_3$): δ 7.84 (d, J = 1.6 Hz, 2H), 7.68 (d, J = 8.0 Hz, 2H), 7.58 (d, J = 1.7 Hz, 4H), 7.44 (dd, J = 8.0, 1.7 Hz, 2H), 7.40 (d, J = 8.2 Hz, 4H), 6.34 (s, 4H), 2.43 (s, 6H). ^{13}C-NMR (101 MHz, $CDCl_3$) δ 196.10 , 173.76 , 140.64 , 138.05 (d, J = 4.4 Hz), 136.85 , 134.86 , 131.97 , 130.62 , 130.53 , 127.67 , 127.26 , 123.25 , 122.99 , 120.04 , 94.04 , 91.61, 32.93. Elemental analysis, exp. (calc., %): C 66.01 (65.90); H 3.38 (3.32); O 4.31 (4.39); S 26.30 (26.39).

(((9,10-bis(dibromomethylene)-9,10-dihydroanthracene-2,6-diyl)bis(ethyne-2,1-diyl))bis(4,1-phenylene))bis(tert-butylsulfide), 5

In a dry 50 mL flask under N_2 were introduced 300 mg of **2** (0.51 mmol) and 1.1 g of PPh_3 (4.1 mmol) and the flask put in an ice bath. 680 mg of CBr_4 (2.1 mmol) were added immediately followed by 25 mL of ice-cold dry DCM dropwise while stirring the formed slurry. After 3 hours the ice bath was removed and the system allowed to rt overnight. The system

was then heated to 40 °C for 2 hours and the hot solution filtered to remove the precipitate (mainly **2**). The solution was preadsorbed on silica and the product purified via column chromatography (SiO_2, hexane/ethylacetate 17:1, Rf= 0.9). A yellow solid was obtained (228 mg, 49% yield). ^{1}H-NMR (400 MHz, $CDCl_3$): δ 8.00 (d, J = 1.6 Hz, 2H), 7.83 (d, J = 8.1 Hz, 2H), 7.55 – 7.41 (m, 10H), 1.30 (s, 18H). ^{13}C-NMR (101 MHz, $CDCl_3$) δ 140.99, 139.88, 138.47, 138.04, 136.36, 134.21, 133.22, 132.81, 130.47, 125.82, 124.81, 94.31, 92.80, 92.76, 49.21, 33.65.

S,S'-(((9,10-bis(dibromomethylene)-9,10-dihydroanthracene-2,6-diyl)bis(ethyne-2,1-diyl))bis(4,1-phenylene)) diethanethioate, ABr

In a dry 50 mL flask under N_2, 80 mg of **5** (0.09 mmol) and 1.38 mL of acetylchloride (19.3 mmol) were dissolved in 28 mL of a 1:1 dry DCM/dry toluene solution. 2.67 mL of a BBr_3 solution 1 M in DCM (2.7 mmol) were added dropwise and the reaction left for 4.5 hours. The mixture was then poured in 150 mL water and 50 mL of DCM were added. The water phase was neutralized using a saturated solution of $NaHCO_3$. The organic phase was extracted with water, dried over Na_2SO_4, and DCM let evaporate. The product was purified using column chromatography (SiO_2, DCM/hexane 4:1, Rf= 0.7) and recrystallized from hexane to obtain yellow cubic crystals suitable for X-ray diffraction (40 mg, 52% yield). ^{1}H-NMR (400 MHz, $CDCl_3$): δ 7.99 (d, J = 1.6 Hz, 2H), 7.83 (d, J = 8.1 Hz, 2H), 7.56 (d, J = 8.3 Hz, 4H), 7.45 (dd, J = 8.1, 1.7 Hz, 2H), 7.40 (d, J = 8.3 Hz, 4H), 2.44 (s, 6H). ^{13}C-NMR (101 MHz, $CDCl_3$) δ 195.97, 140.95, 138.47, 138.15, 136.89, 134.89, 133.28, 132.88, 131.11, 130.48, 126.72, 124.66, 94.39, 92.95, 92.59, 32.96.

(((9,10-bis(diphenylmethylene)-9,10-dihydroanthracene-2,6-diyl)bis(ethyne-2,1-diyl))bis(4,1-phenylene))bis(tert-butylsulfide), 6

In a 50 mL flask equipped with a cooler, 212 mg of **5** (0.24 mmol) and 176 mg of phenylboronic acid (1.44 mmol) were dissolved in 25 mL of toluene and the solution was bubbled

with N_2. 1 mL of a degassed 1:1 EtOH/water solution was added followed by 20 mg of $Pd(PPh_3)_4$ and 265 mg of K_2CO_3. The reaction was kept at refulx for 22 hours. The solvent was rotavaped and the residue dissolved in DCM and extracted with water. The organic phase was preadsorbed on silica and purified over a short column (SiO_2, DCM/hexane 1:3 to pure DCM). The product was finally recrystallized from $CHCl_3$/hexane and obtained as off-yellow needle crystals suitable for X-Ray diffraction (155 mg, 73% yield). ^{1}H NMR (400 MHz, DMSO-d6) δ 7.68 – 7.04 (m, 34H), 1.23 (s, 18H).

S,S'-(((9,10-bis(diphenylmethylene)-9,10-dihydroanthracene-2,6-diyl)bis(ethyne-2,1-diyl))bis(4,1-phenylene)) diethanethioate, APh

In a dry 100 mL flask under N_2, 68 mg of **6** (0.08 mmol) and 1.19 mL of acetylchloride (16.7 mmol) were dissolved in 40 mL of a 1:1 dry DCM/dry toluene solution. 2.30 mL of a BBr_3 solution 1 M in DCM (2.3 mmol) were added dropwise and the reaction left for 4.5 hours. The mixture was then poured in 150 mL water and 75 mL of $CHCl_3$ were added. The water phase was neutralized using a saturated solution of $NaHCO_3$. The organic phase was extracted with water, brine, and dried over Na_2SO_4. The product was purified using column chromatography (SiO_2, $CHCl_3$/hexane 8:1, Rf= 0.8) and finally recrystallized from hexane to obtain an off-white solid. (33 mg, 48% yiled). ^{1}H-NMR (400 MHz, $CDCl_3$): δ 7.50 – 7.21 (m, 32H), 6.97 (d, J = 8.1 Hz, 2H), 6.91 (d, J = 8.1 Hz, 2H), 2.43 (s, 6H). ^{13}C-NMR (101 MHz, $CDCl_3$) δ 196.13, 144.60, 144.43, 144.10, 140.53, 140.29, 136.96, 136.81, 134.72, 133.96, 132.16, 132.10, 131.19, 131.08, 130.75, 130.43, 129.83, 129.65, 122.39, 93.86, 32.94. HR-MS ESI for $C_{60}H_{41}O_2S_2$ calcd mass 857.25425, found 857.25646.

2,6-bis((triisopropylsilyl)ethynyl)anthracene-9,10-dione, 8

In a dry 500 mL flask under N_2, 3.0 g of **1** (8.2 mmol), 4.6 mL of (triisopropylsilyl)acetylene (21 mmol), 250 mg of $Pd(PPh_3)_4$, and 100 mg of CuI were dissolved in 200 mL of dry THF and 10 mL of NEt_3. The solution was heated to 65 °C and left under stirring 16 hours. The

solvent was removed under vacuum and dissolved in $CHCl_3$. The solution was extracted with HCl 1 M and brine, then it was dried over Na_2SO_4 and the solvent removed. The product was finally recrystallized from hexane as an off-white powder (2.1 g, 44% yield). ^{1}H-NMR (400 MHz, $CDCl_3$): δ 8.35 (d, J = 1.6 Hz, 2H), 8.25 (d, J = 8.0 Hz, 2H), 7.84 (dd, J = 8.0, 1.6 Hz, 2H), 1.16 (s, 42H).

((9,10-bis(dibromomethylene)-9,10-dihydroanthracene-2,6-diyl)bis(ethyne-2,1-diyl))bis(triisopropylsilane), 9

In a dry 50 mL flask under N_2 were introduced 900 mg of **8** (1.5 mmol) and 2.9 g of PPh_3 (12.3 mmol) and the flask put in an ice bath. 1.8 g of CBr_4 (5.5 mmol) were added immediately followed by 25 mL of ice-cold dry DCM dropwise while stirring the formed slurry. The system allowed to rt overnight. The solution was filtered and the filtrate preadsorbed on silica. The product was purified over a short silica column (hexane) and obtained as a white solid after the solvent was removed in vacuum (1.3 g, 91% yield). ^{1}H-NMR (400 MHz, $CDCl_3$): δ 7.92 (d, J = 1.7 Hz, 2H), 7.75 (d, J = 8.1 Hz, 2H), 7.38 (dd, J = 8.1, 1.7 Hz, 2H), 1.12 (s, 42H). ^{13}C-NMR (101 MHz, $CDCl_3$) δ 141.04, 138.32, 137.97, 133.60, 133.22, 130.26, 125.23, 108.95, 95.16, 94.21, 21.38, 13.99.

((9,10-di(propan-2-ylidene)-9,10-dihydroanthracene-2,6-diyl)bis(ethyne-2,1-diyl)) bis(triisopropylsilane), 10

In a dry 100 mL flask equipped with a cooler under N_2, 2.1 mL of MeLi 1.6 M in Et_2O (3.4 mmol) and 3.4 mL of B-Methoxy-9-BBN 1M in hexanes (3.4 mmol) were added to 50 mL of dry THF. 653 mg of **9** (0.74 mmol) and 86 mg of $Pd(PPh_3)_4$ were introduced and the system heated to reflux for 10 hours. The solvent was then removed under vacuum and the solid residue extracted with hot hexane. The solution was filtered trhough a plug of silica to obtain the pure product as an off-white solid (332 mg, 72% yield). ^{1}H-NMR (400 MHz, $CDCl_3$): δ 7.47 (s, 2H), 7.27 (s, broad, 4H), 2.12 (s, 6H), 2.07 (s, 6H), 1.13 (s, 42H).

2,6-diethynyl-9,10-di(propan-2-ylidene)-9,10-dihydroanthracene, 10.1

In a 250 mL flask, 332 mg of **10** (0.53 mmol) were dissolved in 125 mL of THF and the solution was put in an ice bath. 1.34 mL of tetrabutylammonium fluoride 1M in THF (containing 5% vv. water, 1.34 mmol) were added dropwise and the reaction was allowed to room temperature overnight. The solution was dried with Na_2SO_4 and preadsorbed on silica to be purified over a short silica column (hexane). The product was obtained as a white solid (160 mg, 99% yield). ^{1}H-NMR (400 MHz, $CDCl_3$): δ 7.51 (s, 2H), 7.32 (s, 4H), 3.05 (s, 2H), 2.11 (s, 6H), 2.08 (s, 6H). ^{13}C-NMR (101 MHz, $CDCl_3$) δ 141.80, 141.12, 133.78, 133.67, 133.23, 131.41, 130.34, 121.21, 86.76, 79.26, 34.26.

S,S'-(((9,10-di(propan-2-ylidene)-9,10-dihydroanthracene-2,6-diyl)bis(ethyne-2,1-diyl))bis(4,1-phenylene)) diethanethioate, AMe

In a dry Schlenk under N_2, 145 mg of **10.1** (0.47 mmol) and 386 mg of **IPhSAc** (1.39 mmol) were dissolved in 10 mL mL of dry THF. 60 mg of $Pd(PPh_3)_4$ and 32 mg of CuI were added followed by 1.5 mL of freshly distilled NEt_3 and the reaction was left at 50 °C overnight. The solution was poured in 100 mL DCM and 50 mL water under stirring and HCl 6 M was added dropwise until neutrality was reached. The organic phase was then extracted with water, dried over Na_2SO_4, and preadsorbed on silica. The product was purified via column chromatography (SiO_2, hexane:ethylacetate 5:1, Rf=0.4) and recrystallized from hexane. It was obtained as a white solid (95 mg, 33% yield). ^{1}H-NMR (400 MHz, $CDCl_3$): δ 7.57 – 7.52 (m, 6H), 7.41 – 7.31 (m, 8H), 2.43 (s, 6H), 2.15 (s, 6H), 2.11 (s, 6H). ^{13}C-NMR (101 MHz, $CDCl_3$) δ 193.47, 143.99, 138.98, 138.57, 134.18, 132.13, 130.99, 130.70, 130.66, 130.46, 128.32, 127.77, 124.75, 119.34, 30.26, 23.05. Elemental analysis, exp. (calc., %): C 78.41 (78.91), H 5.18 (5.30), O 5.65 (5.26), S 10.76 (10.53). HR-MS APCI for $C_{40}H_{33}O_2S_2$ calcd 609.19165, found 609.19429.

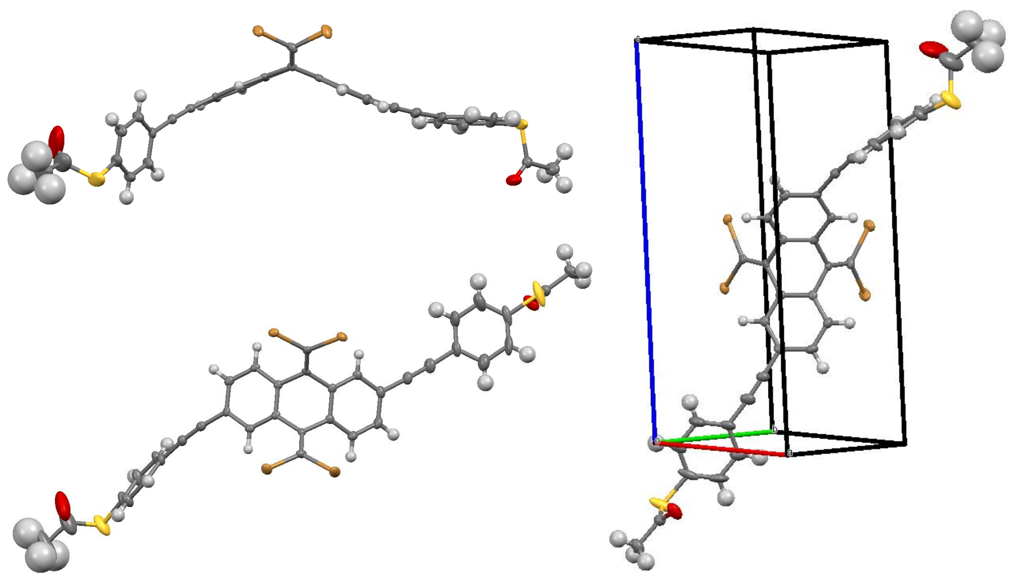

Figure S2: Crystal structure of **ABr**.

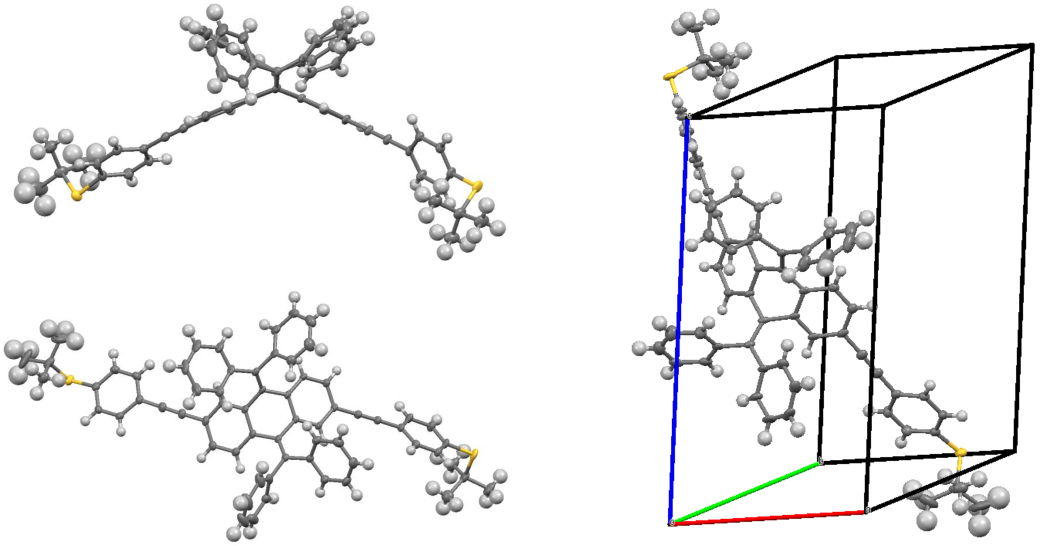

Figure S3: Crystal structure of **6**

Table S1: Crystallographic data for **ABr** and **APh**.

Compound	**ABr**	**APh**
Chemical formula	$C_{36}H_{20}Br_4O_2S_2$	$C_{64}H_{52}S_2$
M_r	868.28	885.17
Crystallographic System	triclinic	triclinic
Color, Habit	colorless, platelet	colourless, needle
Space Group	P -1	P -1
a (Å)	7.9990(5)	10.0873(7)
b (Å)	12.0346(8)	16.6335(12)
c (Å)	17.8718(11)	19.3200(19)
α (deg)	96.267(2)	69.741(3)
β (deg)	90.136(3)	87.735(3)
γ (deg)	108.018(2)	81.507(3)
V ($Å^3$)	1625.09(18)	2465.1(4)
Z	2	2
ρ_{calc} (g/cm^3)	1.774	1.193
μ (Mo K$^\alpha$, (cm^{-1})	0.71703	0.71073
F(000)	848	936
T (K)	100(2)	100(2)
θ range (deg)	2.74 - 27.19	3.018 - 26.428
Data collected (h,k,l)	-10:10, -15:15, -22:22	-12:12, -15:17, -24:24
no. of reflections collected	35850	31372
no. of independent reflections	9986	10012
observed reflections	7160 ($F_o \geq 2\sigma(F_o)$)	7103 ($F_o \geq 2\sigma(F_o)$)
R(F) (%)	6.44	5.73
wR(F^2) (%)	9.12	12.78
GooF	1.020	1.022
Parameters refined	399	601
restraints	20	0

2.1 Preparation of the other derivatives

We were able to synthesize the aforementioned compounds, but the others — **A(CH$_2$)**, **AF**, **A(All)**, **A(Alk)** — presented in Figure 1 in the Main Text remain a challenge. As we will discuss in this section this is due mostly to the limited stability of these compounds or their intermediates.

In the case of **A(CH$_2$)** the simplest all-carbon derivative of **AQ**, proposed by Valkenier *et al.*,[S3] all our synthetic attempts were unsuccessful. While **1**, **2**, nor anthraquinone did not react in Wittig conditions, the use of other methods (involving for example titanium-carbene complexes) yielded in black residue that showed no unequivocal trace of the desired products. This is probably due to the limited stability of the 9,10-anthraquinonedimethane core which was reported to readily polymerize unless kept in extremely diluted solutions.[S4] The same reactions performed on diphenylketone yielded the expected products, emphasizing the different reactivity of quinoid structures compared to ketones.

Limited stability was also observed in the case of **AMe**. As specified in the main text, we tried a procedure similar to that for the preparation of **ATTF** but no product could be obtained from the replacement of the StBu groups with SAc in **11** using $TiCl_4$ as shown in in Figure S4). On top of that, when compared to the other compounds, **AMe** tend to become dark within days if not kept in the dark. We believe that this limited stability has the same thermodynamic origin of the aforementioned **A(CH$_2$)**, with the difference that in this latter case the methyl groups slow down the kinetics.

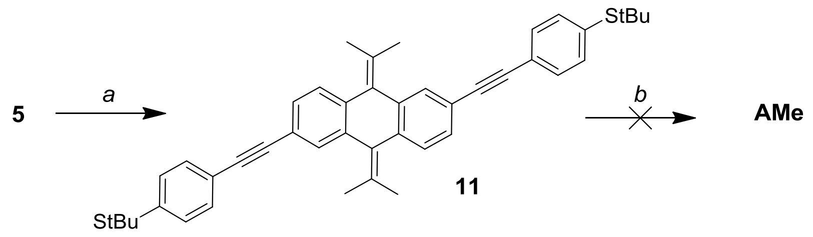

Figure S4: Reaction scheme for a proposed synthesis of **AMe**: a) B-MeO-9-borabycyclononane, MeLi, $Pd(PPh_3)_4$, THF; b) AcCl, $TiCl_4$, DCM.

As for **A(CH$_2$)**, we were not able to perform the synthesis of its fluorinated analogue,

AF. While we were able to react anthraquinone core in a Reformatsky-type reaction using 2,2-bromo-difluoro-ethylacetate (**12**), elimination of the ester group in acid conditions did not produce the expected difluorovinyl functionality but a dark residue from which we could only recover starting material (Figure S5. Our hypothesis is that, if a difluorovinyl group is formed during the reaction conditions, it can follow the same face as the the methylene derivative of anthraquinone core previously reported.

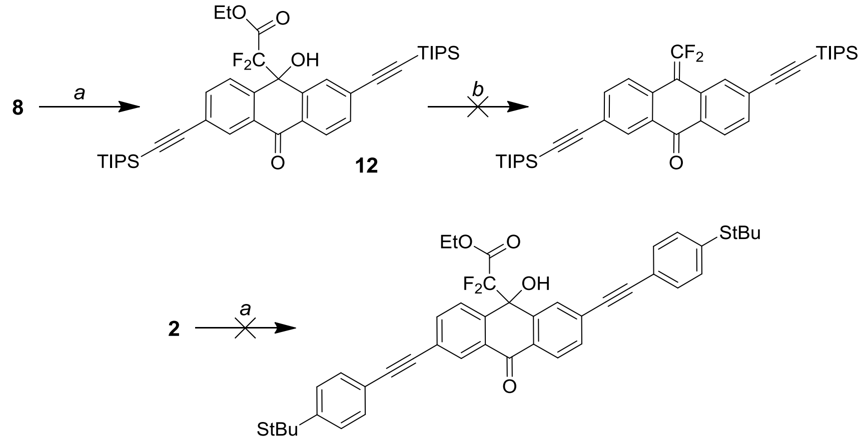

Figure S5: Reaction scheme for a proposed synthesis of **AF**: a) Ethyl bromodifluoroacetate, $ZnCl_2$, THF; b) AcOH, Ac_2O.

Among the all-carbon analogues of **AQ**, **A(All)** is of particular interest as it should retain the same flatness of the core. We tried the synthesis by reduction of the correspondent propargyl alcohol (**13**) to the allene moiety but it was unsuccesful as the aromatic core rearranges to anthracene instead. If the TMS groups are removed from **13**, the core rapidly rearranges to anthraquinone by loosing acetylene.

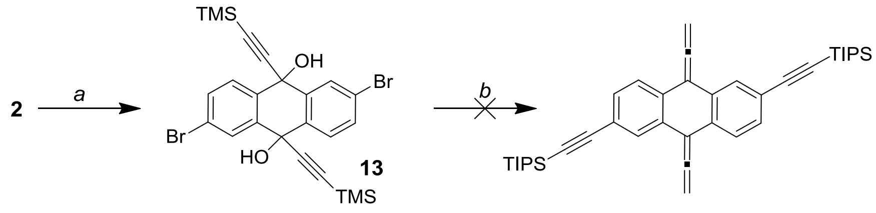

Figure S6: Reaction scheme for a proposed synthesis of **A(All)**: a) lithium TMS-acetylide, THF; b) *o*-nitrobenzenesulfonylhydrazide, triflate Ag(I), triflic acid, nitromethane.

Finally, considering the reactivity of **5**, we decided to prepare from it **A(Alk)**, the

all-carbon equivalent of **TCNAQ**, using TMS-protected ethynyl groups. Anyway, such compound was found not to be stable and turned darker even when kept in the dark under nitrogen atmosphere.

2.1.1 Synthetic procedures

ethyl 2,2-difluoro-2-(9-hydroxy-10-oxo-2,6-bis((triisopropylsilyl)ethynyl)-9,10-dihydro anthracen-9-yl)acetate, 12

In a dry 50 mL flask under N_2, 280 mg of **8** (0.49 mmol) and 0.1 mL of ethyl bromodifluoroacetate (0.79 mmol) were dissolved in 25 mL of dry THF and the solution placed in an ice bath. 0.8 mL of $ZnCl_2$ 0.9 M in hexanes (0.72 mmol) were added dropwise and the system was allowed to roo tmeperature overnight. 15 mL of HCl 1 M were added and the organic phase was extracted with NH_4Cl. It was then dried over Na_2SO_4. The product was obtained as an orange oil (255 mg, 80% yield). ^{1}H-NMR (400 MHz, $CDCl_3$): δ 8.22 (d, J = 1.7 Hz, 1H), 8.09 (d, J = 8.1 Hz, 1H), 7.97 (d, J = 1.5 Hz, 1H), 7.81 (d, J = 8.2 Hz, 1H), 7.70 (dd, J = 8.2, 1.8 Hz, 1H), 7.52 (dd, J = 8.1, 1.5 Hz, 1H), 2.48 (broad, 1H), 2.02 (q, J = 7.4 Hz, 2H), 1.15 (d, J = 3.3 Hz, 42H), 0.34 (t, J = 7.4 Hz, 3H).

2,6-dibromo-9,10-bis((trimethylsilyl)ethynyl)-9,10-dihydroanthracene-9,10-diol, 13

In a dry 100 mL flask under N_2, 1.15 mL of TMS-acetylene (8.2 mmol) were dissolved in 50 mL of dry THF and the solution moved in an ice/NaCl bath. 5.05 mL of *n*-butyllithium 1.6 M in hexanes (8.1 mmol) was added slowly and the system left for 1 hour. 500 mg of **1** (1.37 mmol) were added and the system allowed to rt ovetrnight. The solution was extracted with NH_4Cl aq. sat., dried over Na_2SO_4, and adsorbed on neutral alumina. It was purified through a small column (neutral Al_2O_3, hexane/ethylacetate 1:1). A yellow oil was obtained (336 mg, 44% yield). ^{1}H-NMR (400 MHz, $CDCl_3$): δ 8.16 (d, J = 1.9 Hz, 2H), 7.87 (d, J = 8.3, 2H), 7.54 (dd, J = 8.4, 2.0 Hz, 2H), 3.78 (s, 2H), 0.15 (s, 18H).

(((9,10-di(propan-2-ylidene)-9,10-dihydroanthracene-2,6-diyl)bis(ethyne-2,1-diyl))bis(4,1-phenylene))bis(tert-butylsulfane), 11

In a dry 50 mL flask equipped with a cooler under N_2, 0.25 mL of MeLi 1.6 M in Et_2O (0.4 mmol) and 0.40 mL mL of B-Methoxy-9-BBN 1M in hexanes (0.4 mmol) were added to 25 mL of dry THF. After 10 minutes, 82 mg of **5** (0.1 mmol) and 10 mg of $Pd(PPh_3)_4$ were introduced and the system heated to reflux for 1 hour. The solvent was then removed under vacuum and the oily residue dissolved in DCM and filtered. The solution was absorbed on silica and purified through column chromatography (SiO_2, hexane/ethylacetate 10:1). The product was obtaine das an off-white solid (61 mg, 96% yield). ^{1}H-NMR (400 MHz, $CDCl_3$): δ 7.59 – 7.42 (m, 8H), 7.41 – 7.29 (m, 6H), 2.15 (s, 6H), 2.11 (s, 6H), 1.30 (s, 18H). ^{13}C-NMR (101 MHz, $CDCl_3$) δ 138.88, 138.57, 137.21, 131.45, 130.92, 130.61, 128.54, 128.42, 128.26, 127.76, 123.84, 119.48, 91.45, 88.38, 46.44, 30.98, 29.68.

3 SAM preparation and characterization

3.1 SAMs formation

All the SAMs were prepared under nitrogen atmosphere. For Au on mica substrates, the metal surfaces (1x1 cm, 200 nm thick Au, obtained from Phasis, Switzerland) were incubated for two days in a 0.5 mM solutions of the different molecular wires in dry chloroform to which 0.3 mL of distilled triethylamine were added according to a know procedure.[S1] The SAMs were finally rinsed with dry chloroform and let dry for 20 minutes before the measurement. DCM instead of chloroform was used in the case **AQ** as it gave less dispersion in the data. The current values between the two SAMs did not differ significantly as can be seen in Figure S7.

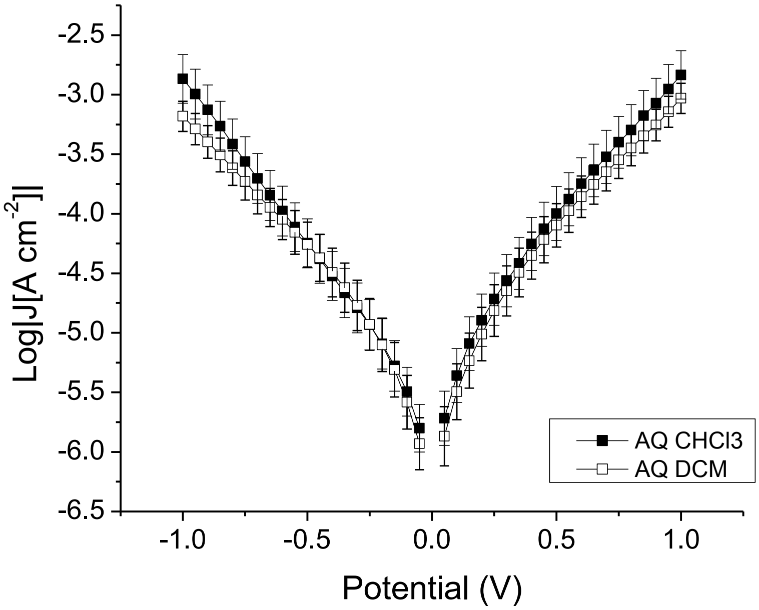

Figure S7: $Log|J|$ vs. V plot for Au^{Mica}/**AQ**//EGaIn junctions comprising SAMs grown from $CHCl_3$ (solid squares) or DCM (open squares). Error bars represent confidence intervals ($\alpha = 0.05$).

For template-stripped metal substrates (Au^{TS}), the SAMs were formed by incubating a 1x1 cm template-stripped metal surface (100 nm-thick) overnight in 3 mL of a 50 µmol solution of the compound in dry toluene followed by addition of 0.05 mL of 17 mM diazabicycloundec-7-ene (DBU) solution in dry toluene 1.5 hours prior the measurement, according to a known

procedure.[S5] The substrates were then rinsed with ethanol and let to dry for 30 minutes before performing the measurements. SAMs of **AC** on Au^{TS} were prepared and measured elsewhere.[S6] The difference in nthe morphology of the two substrates is shown in Figure S8 and S9.

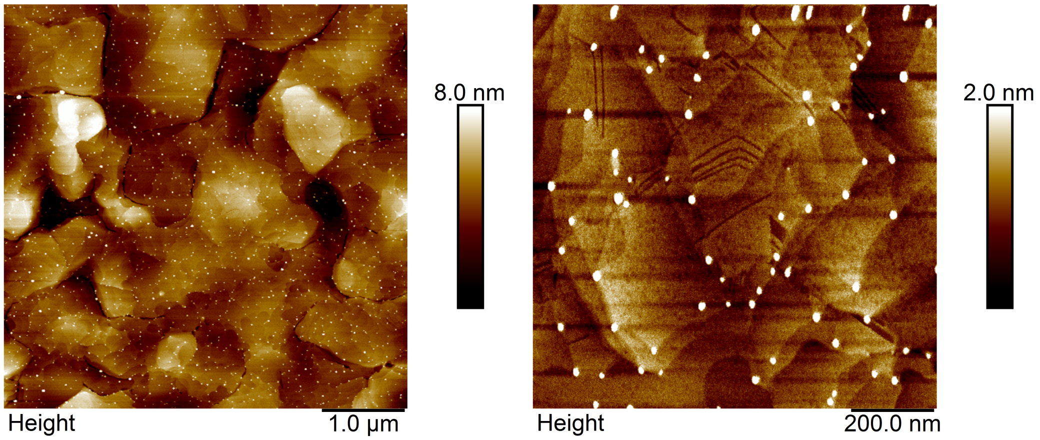

Figure S8: AFM height profiles of Au^{mica} substrate scanned at 5 μm (left) and 1 μm(right).

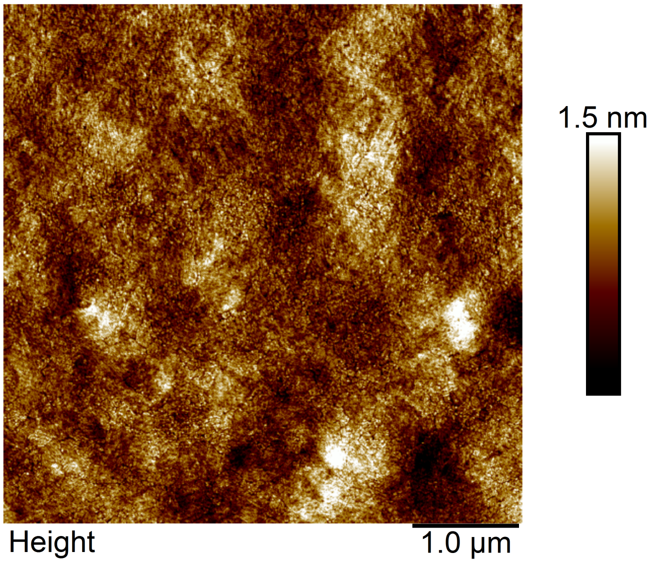

Figure S9: AFM height profiles of Au^{TS} substrate scanned at 5 μm.

3.2 Spectroscopic characterization of the SAMs

The **TCNAQ** monolayer was characterized in detail somewhere else.[S2] Representative of the entire series, some of the films addressed in this study were characterized by synchrotron-based X-ray photoelectron spectroscopy (XPS) and near-edge X-ray absorption fine structure (NEXAFS) spectroscopy to verify their SAM character and to determine their basic parameters. The measurements were performed at the dipole-magnet HE-SGM beamline at the German Synchrotron Radiation Facility, BESSY II in Berlin. The description of the experimental setup, the relevant experimental parameters, and the details of the data evaluation procedure can be found elsewhere.[S7] The data for the **AQ** and **TCNAQ** films, suggesting their distinct SAM character can be found in our previous publications[S1,S7] and in the next section respectively. The data for the **APh**, **ABr**, and **ATTF** films are presented below. The S 2p XP spectra of these films in Figure S10a exhibit characteristic signals of thiolate at ~162.0 eV for S 2p3/2 (**1**) and unbound SAc groups at 163.9 – 164.1 eV for S 2p3/2 (**2**), with much higher intensity of the latter signals, because of the differences in the attenuation for the buried (thiolate) and terminal (SAc) groups.[S8,S9] These spectra suggest that the molecules are indeed assembled upright, in the SAM fashion, with one of the terminal SAc groups bound to the substrate and another one exposed to the SAM-ambient interface, where it can be contacted by the EGa-In electrode. The doublet at 163.9 – 164.1 eV is especially strong for the **ATTF** SAM because the signal of the unbound SAc groups (**2**) overlaps with the contribution of the sulfur atoms in the 1,3-dithiole moieties (**3**), appearing at the same binding energy. In addition, there is a weak signal of atomic sulfur at 161.0 eV for S 2p3/2 (**4**) in some of the spectra; such a minor contamination is frequently observed in the S 2p XP spectra of thiolate SAMs.[S9]

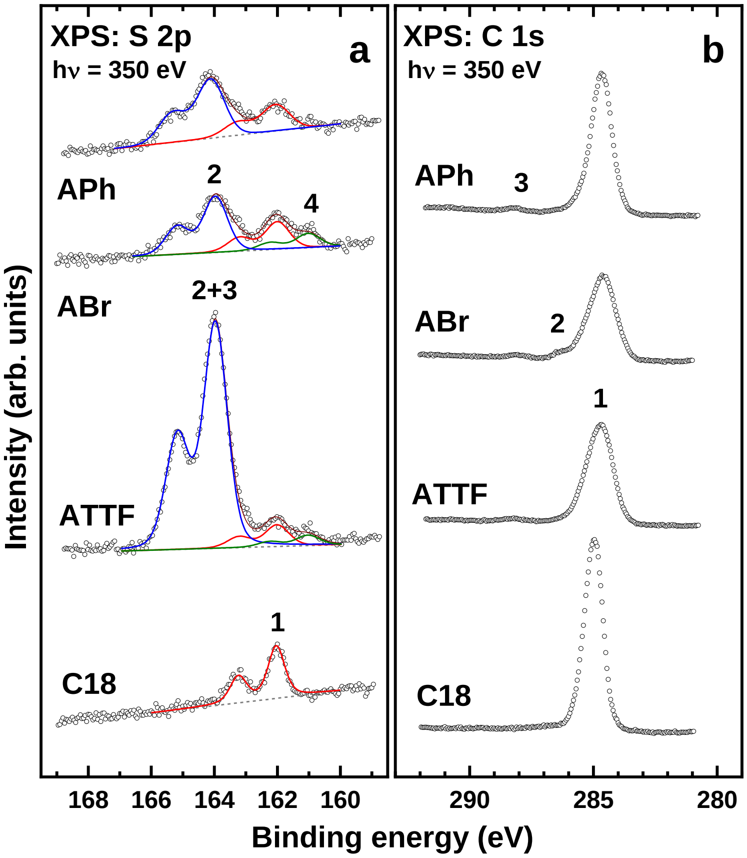

Figure S10: S 2p (a) and C 1s (b) XP spectra of the **APh**, **ABr**, and **ATTF** SAMs as well as reference **C18** monolayer. The spectra were acquired at a photon energy of 350 eV. The S 2p spectra are decomposed into individual doublets, drawn by different colors and marked by numbers (see text for details); individual peaks in the C 1s spectra are marked by numbers as well (see text for details).

Based on the intensity of the thiolate signal, packing density of the **APh**, **ABr**, and **ATTF** SAMs was calculated, taken the S2p/Au4f intensity ratio as a measure and the octadecanethiolate SAM (**C18**) with a known packing density of 4.63×10^{14} molecules/cm^2 [S10] as the reference. The resulting values are compiled in Table S2. They are quite similar with respect to each other but somewhat smaller than that for the reference **C18** SAM, which is understandable in view of the bulky character of their backbone core.

Table S2: Packing density and effective thickness of the **APh**, **ABr**, and **ATTF** SAMs derived from the XPS data, along with the reference values for the **C18** monolayer.

Compound	Packing density (10^{14} molecules/cm^2)	Thickness (Å)
**C18**	4.63	20.9 ± 0.2
**APh**	2.9 ± 0.2	20.3 ± 0.5
**ABr**	2.7 ± 0.2	18.0 ± 0.5
**ATTF**	2.7 ± 0.2	17.9 ± 0.5

The C 1s XP spectra of the **APh**, **ABr**, and **ATTF** SAMs in Figure S10b are dominated by the strong signal of the molecular backbone at 284.5 –284.7 eV (**1**) accompanied by a shoulder at ∼286.3 eV (**2**) and a weak peak at 288.1 eV (**3**). The shoulder, observed in the spectrum of the **ABr** SAM only, stems from the carbon atoms bound to Br, with the latter atoms being clearly observed in the respective Br 3d XP spectrum (Figure S11). The weak peak is most likely related to a minor C=O or COOH contamination,[S11] which is hardly avoidable in view of the steric constrains upon the molecular assembly of such complex molecules with a bulky backbone core.

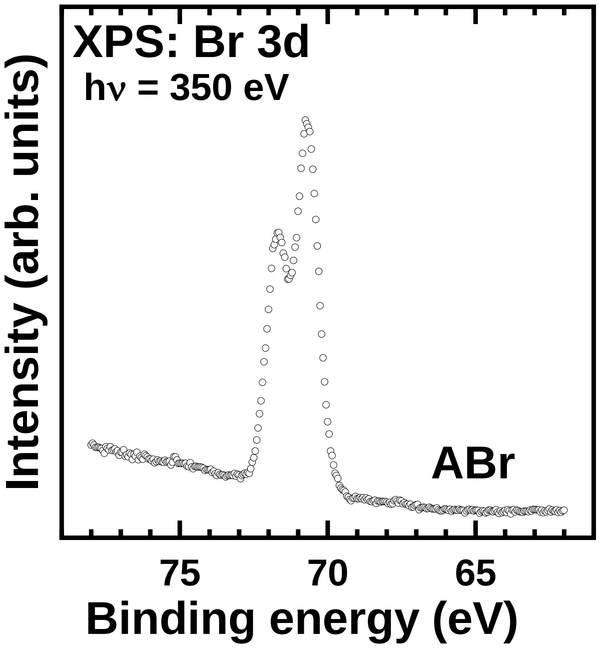

Figure S11: Br 3d XP spectrum of the **ABr** SAM. The spectrum was acquired at a photon energy of 350 eV.

Based on the C1s/Au4f intensity ratio, the effective thickness of the **APh**, **ABr**, and

ATTF SAMs was evaluated, taking the **C18** SAM with a known thickness of 20.9 ± 0.2 Åas the reference. The resulting values are compiled in Table S2. They correspond to monomolecular films (as expected) but are smaller than the lengths of the respective molecules, which suggest that the molecules in the SAMs are tilted. Additional information is provided by the NEXAFS data. The C K-edge NEXAFS spectra of the **APh**, **ABr**, and **ATTF** SAMs acquired at an X-ray incidence angle of 55° are presented in Figure S12. At this particular orientation the spectra are exclusively representative of the electronic structure of the monolayers.[S12] The spectra exhibit the characteristic shape and the characteristic absorption resonances of the oligo(phenyleneethynylene) (OPE)[S13–S15] and oligophenyl[S16] compounds, above all the slightly asymmetric, joint $\pi^*/\pi_1{}^*$ resonance at ~285.0 eV (**1**). This resonance is particular pronounced in the spectrum of the **APh** SAM, because of the strong contribution of the 'side' phenyl rings. The resonances at 287.3 eV (**2**) and 288.9 eV (**3**) have most likely the Rydberg and $\pi_2{}^*$ character, respectively, while the broader resonances at higher photon energies have the σ^* character.[S13–S16] Significantly, the intensity of the resonance at 288.9 eV (**3**), which can contain a contribution of carboxyl,[S11,S17] is comparably small, which means, in accordance with the XPS data (see above), a minor character of this contamination.

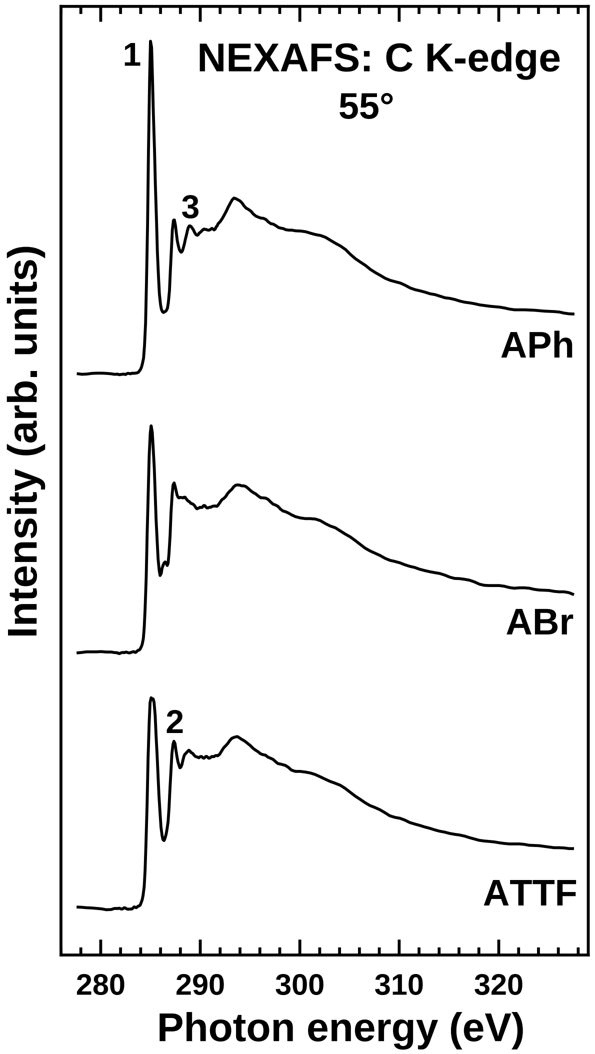

Figure S12: C K-edge NEXAFS spectra of the **APh**, **ABr**, and **ATTF**SAMs acquired at an X-ray incidence angle of 55°. The most prominent resonances are marked by numbers (see text for details).

The NEXAFS spectra of the **APh**, **ABr**, and **ATTF** SAMs exhibit quite small linear dichroism (dependence of the resonance intensity on the angle of X-ray incidence) which can both mean a disordered film or a molecular inclination close to a tilt angle of 35°.[S12] Evaluation of the entire set of the NEXAFS data, based on the standard formalism for a vector-like orbital (π_1* in the given case),[S12] resulted in average molecular tilt angles of 33.5°,35.5°, and 31.0° for the **APh**, **ABr**, and **ATTF** SAMs respectively. Note that these values should be considered as tentative only, reflecting an upright (even though with a tilt) molecular orientation in the SAMs studied.

4 Electrical characterization of molecular junctions comprising SAMs

4.1 Large area EGaIn junctions

All the electrical characterizations were performed in a controlled nitrogen atmosphere containing 1-3% O_2 and RH$<$ 15%. The details of such EGaIn setup are described elsewhere.[S5] For each compound, both on Au^{Mica} and Au^{TS}, 3 or 4 substrates were measured. For each sample, we measured 15 Au/SAM//EGaIn junctions (5 scans from $0V \rightarrow 1V \rightarrow -1V \rightarrow 0V$, steps of 0.05 V, 0.1 s delay between steps) for a total of at least 75 traces per sample. A new EGaIn tip was prepared every 5 junctions. Junctions that shorted at any point during the scans were considered failed junction for the yield calculations. The number of traces measured for each molecule/substrate combination is reported in S3. Data for **AC** on Au^{TS}, **AQ** on Au^{Mica}, and **TCNAQ**on Au^{TS} and Au^{Mica} were collected and analyzed in references S6, S7, S2 respectively.

4.2 Small area CP-AFM junctions

CP-AFM $I - V$ measurements were performed on a Bruker AFM Multimode MMAFM-2 equipped with a PeakForce TUNA Application Module (Bruker). The SAMs were contacted with a Au-coated silicon nitride tip with a nominal radius of 130 nm (NPG-10, Bruker; tip A, resonant frequency = 65 kHz, spring constant = 0.35 N/m; tip B, resonant frequency = 23 kHz, spring constant = 0.12 N/m; tip C, resonant frequency = 56 kHz, spring constant 0.24 N/m; tip D, resonant frequency = 18 kHz, spring constant 0.06 N/m; tip A was chosen in this work) in TUNA mode. The AFM tip was grounded and all samples were prepared on Au^{TS}/Au$mica$ and biased from -1.5 V to +1.5 V and from +1.5 V to -1.5 V (TCNAQ and APh were measured from -1.0 V to +1.0 V) to record the I-V curves: a max of 10 trace/retrace cycles per junction were captured and each trace contains 512 data points.

The top electrode was removed from SAMs and reapplied between junctions.

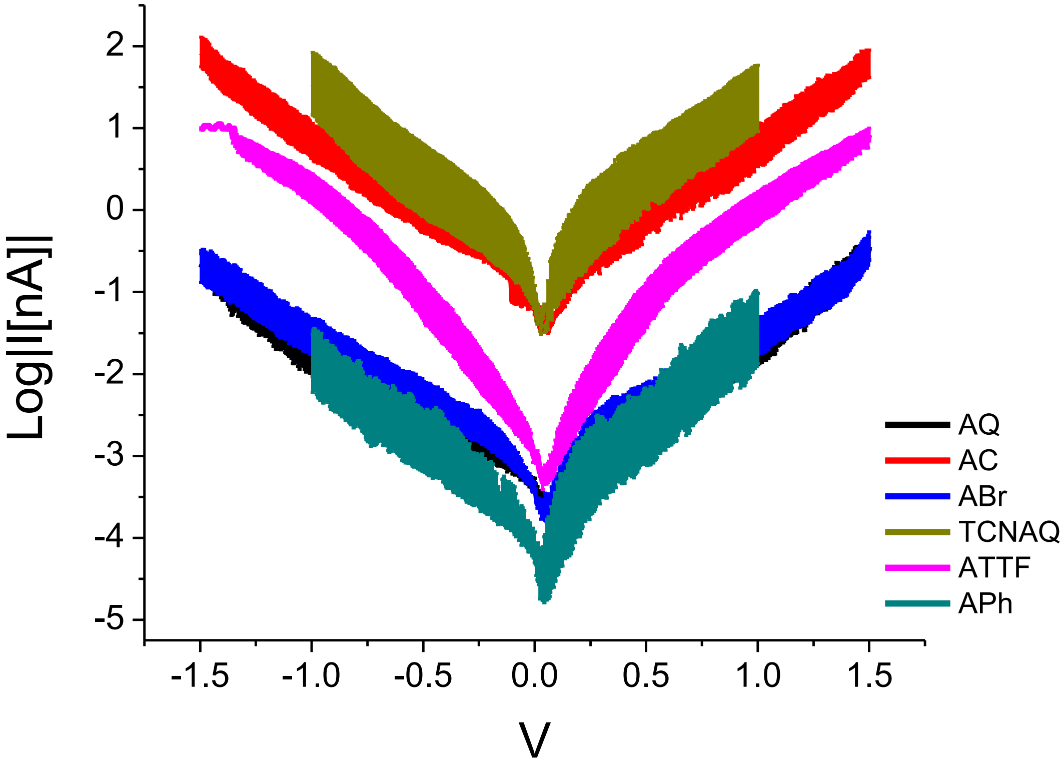

Figure S13: Electrical characterization of tunnelling junctions obtained as $Au^{Mica}/SAM/Au^{CP-AFM}$ comprising **AQ** (black), **AC**(red), **APh** (cyan), **ABr** (blue), **ATTF** (pink), and **TCNAQ** (yellow). Error bars are confidence intervals ($\alpha = 0.05$).

4.3 Process of J-V data

These data were parsed in a "hands-off" manner using Scientific Python to produce histograms of J for each value of V, the associated Gaussian fits (using a least-squares fitting routine) as described elsewhere.[S6] The same raw data were used to produce the NDC plots. Low bias conductance values were obtained from a linear fit of the $J - V$ plots considering the data points between -0.1 V and +0.1 V.

Table S3: Number of traces analyzed per molecule per substrate/measurement condition.

	Au^{Mica}	Au^{TS}	CP-AFM
AQ	220 [S7]	-	469
AC	355	490 [S6]	509
APh	295	225	109
ABr	225	225	1343
TCNAQ	225	225	799
ATTF	225	225	1248

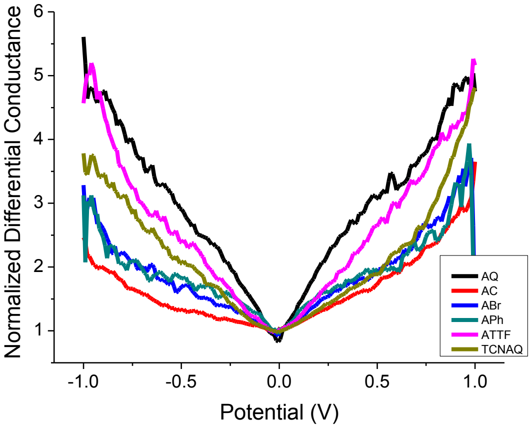

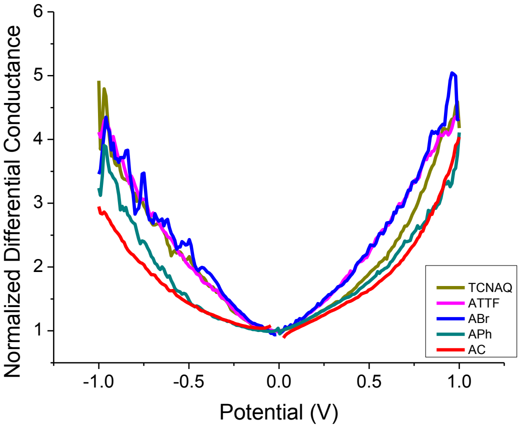

Figure S14: Mean values of NDC vs. V for Au^{Mica}/SAM//EGaIn (left) and Au^{TS}/SAM//EGaIn (right) junctions.

5 Single molecule conductance measurement

The STM break-junction experiments were performed with a Molecular imaging system using PicoScan software. We used a commercial Au on mica substrate (1x1 cm, Phasys, Switzerland) and a gold tip similar to that described in elsewhere.[S18] Prior to each experiment, the substrate was briefly annealed using a hydrogen flame. The STM tip was prepared by cutting a 0.25 mm gold wire (99.999%). The STM cell was cleaned with piranha solution (98% H_2SO_4:30% H_2O_2=3:1 v/v) and then sonicated three times in MilliQ water. We carried out the conductance measurements in a 0.1 mM mesitylene solution of the different compounds. Prior to the measurement, a small amount of NEt_3 was added into solution in order to cleave the thioacetate group.[S19]

Prior to the STM break junction measurement, the quality of the substrate was checked by scanning its surface in STM mode. Clear images and sharp atomic steps usually indicates a clean substrate and a sharp tip. After ensuring the tip and Au/Mica substrate were in good conditions, the STM feedback loop was turned off. Then the tip was precisely driven by the piezo, leading to a repeatedly in and out of contact with the substrate. During such movements, the molecules may bridge both the tip and the substrate electrodes. Meanwhile, current versus tip travel time ($I(s)$) curves are recorded. Single molecular conductance is determined by the current plateau as well as the conductance histogram constructed from large number of individual events.

Figure S15 shows the results of STM break-junction studies for **AC**, **APh**, **ABr**, **ATTF**, **TCNAQ**, and **AQ**. The data concerning **AC**, **AQ**, and **TCNAQ** are discussed somewhere else.[S2] All the traces are recorded when the STM tip was pulled away from the substrate and under the bias voltage of $0.1V$. The $I(s)$ traces are presented in the left part of Figure S15 with arbitrary x-axis offsets. The dramatic conductance drop associated with the tip stretching suggests a decrease in the number of molecules involved in the junctions. The last conductance plateau is usually taken as the single molecular conductance. Higher conductance steps (as found for example in the case of **AC** in Figure S15) suggest that double

molecules may contribute to the conductance of the junctions. To determine the conductance of the single molecules, 5000 current-distance traces are acquired for statistical studies. The conductance maximum of the peaks for the different compounds is reported in Table S4 together with the number of selected traces.

Table S4: Number of selected traces and values of conductance for STM-BJ experiments.

	Number of selected traces	$Log(G/G_0)$
**AC**	710	-4.3
**ABr**	429	-4.7
**APh**	113	-4.6
**ATTF**	360	-4.9
**TCNAQ**	350	-5.1
**AQ**	775	-5.3

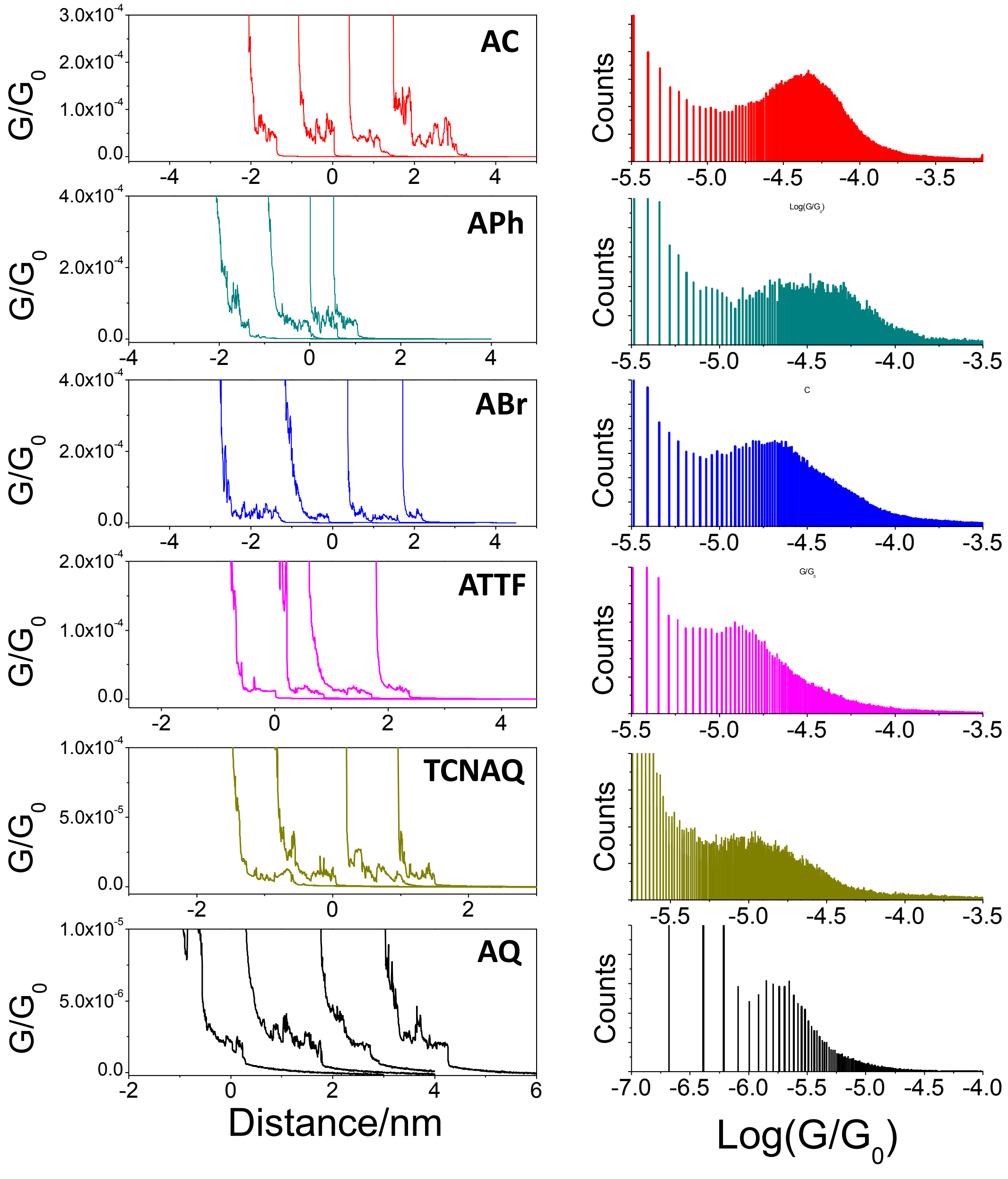

Figure S15: Results for STM-BJ measurements for the different anthraquinoid wires: example of traces (left), distribution of plateau conductance values as $Log(G/G_0)$ (right).

6 Calculations

6.1 Tight-Binding/Hückle Model

An effect of destructive QI in tunnelling junction is the lowering of the overall conductance of a junction due to specific spacial arrangements of atoms and bonds in the molecules. This usually translate in a sharp and narrow dip in the transmission probability vs. energy of the tunnelling electrons plot, which should translate in a lowering tunnelling rate for the junction. Many factors can affect the position of the dip, such as the conjugation path, the relative position of the atoms in the molecule, the electronegativity of the elements, *etc. etc.* In the main text, we reported the transmission probability for our anthraquinoid series in Figure 1. This result was obtained calculating the transport curves from DFT-derived Hamiltonians (see Section 6.2.4) which take in account all the interactions between the atomic orbitals in the molecule and the leads: when functionalities characterized by different electronegativity are present on the molecule, the energy of the feature could fall outside the HOPS-LUPS gap no QI dip is seen.

Here we show how, using a much simpler 2D through-bound model to perform transport calculation, destructive QI is predicted from considerations on the molecular skeletons in most cases. For simplicity, all the atoms were approximated by a single Slater orbital of the same kind whose energy was set to 0 eV, and the coupling energy from the orbital overlap was set to −1 eV for the nearest neighbors (*i.e.*, two sp^2 atoms connected by a bond). The leads, consisting in infinite Au chains, were coupled to the previously mentioned two carbon atoms connected to the rings with a coupling magnitude of 0.5 eV. In such model no distinction are made between the identity of the atoms (*e.g.*, **AQ** and $\mathbf{A(CH_2)}$ will be defined by the same 2D nodes, just like **TCNAQ** and **A(Alk)**). The results of a charge transport calculation on the core of these model systems obtained using the GOLLUM software package, can be observed in Figure S16. Changing the orbital site energy or the coupling energy affect the secondary features of the plots, but not the position of the dip at 0 eV. Although different

molecular skeletons affect the shape of the dip, the latter is always present except in the cases of **AC** (which is linearly conjugated) and **A(All)**. The reason why the latter behaves - for magnitude and shape - like **AC** as to do with an artifact of the model just introduced: when only the connection between the atoms are considered, no distinction can be made between **A(All)** and a fully conjugated anthracene-core wire with two ethynyl substituents in position 9 and 10;the software is evidently more prone on picking on the second one. With some chemical insight one could decide not to account for the terminal atom of the allene moiety, reducing this case to that of $\mathbf{A(CH_2)}$ (and **AQ**), but even with this approach we are making a drastic approximation by treating in the same way the sp^2 carbon of the methylene in $\mathbf{A(CH_2)}$ and the the *sp* carbon in the middle of the allene group, while at the same time disregarding the terminal carbon which also has available *p*-orbitals. These observations show that the tight-binding model, despite being very simple and elegant, is not useful to describe this specific case. When the same transport calculations are performed on the Hamiltonians obtained via DFT, the differences between **A(All)** and **AC** becomes striking, meaning that this latter method can pick on structural and electronic differences despite the atoms connections are identical. In Section 6.2.4 we explore whether geometrical or electronic considerations dictate the transport characteristics in **A(All)**.

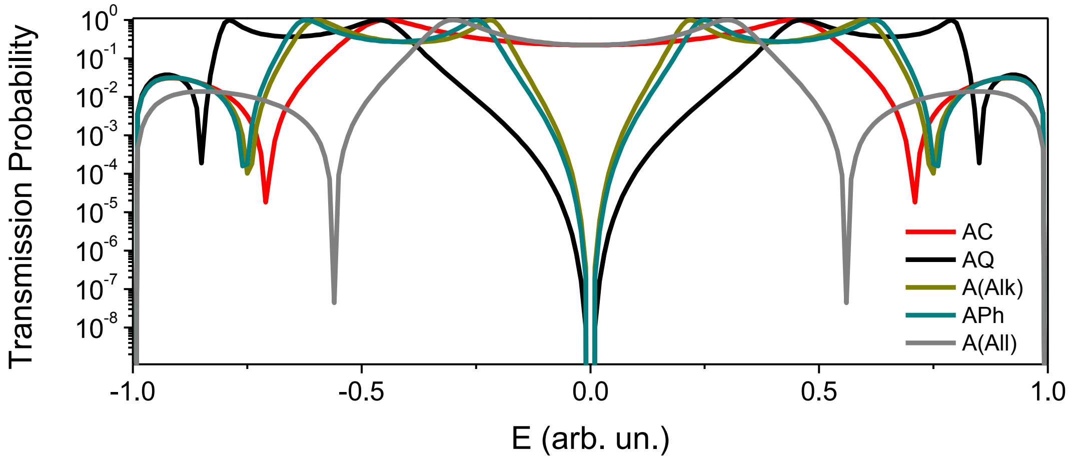

Figure S16: Transmission probability vs. energy of tunnelling electron of 2D-nodes grids corresponding to the molecular skeleton of the anthraquinoid series.

6.2 DFT simulations

We used ORCA[S20,S21] software package for quantum mechanical calculations to perform the Density Functional Theory (DFT) calculations on single molecules and single molecular junctions. We used ARTAIOS-030417 software package for transport calculations, to generate transmission probability spectra.[S22,S23] The procedure is described below step-by-step.

6.2.1 Molecular Geometry Optimization

We minimised the geometries of the molecules included in this study terminating with thiol groups on both ends using the ORCA software package.[S20,S21] We used the default Ahlrichs split-valence $def2 - SVP$ basis sets (ORCA option *Acc-Opt*, that calls the BP functional) with tight SCF and geometry convergence criteria.[S24] The energy of the optimized gas-phase geometry obtained in this calculation was calculated in the next step. The angles ϕ reported in Table 1 in the main text were calculated as angle between the centroids of the benzene ring and the mean point between the position 9 and 10 of the anthraquinoid core as depicted in Figure S17. As angle ϕ increases, also the angle between the hypothetical plane of the molecule (as it was flat) and the lateral double bonds increase with a similar trend.

6.2.2 Single Point Gas-Phase Energy Calculations

We used the ORCA package also for calculating the gas-phase energies for all the molecules. We used the optimized geometries to calculate the single-point gas-phase energies using $B3LYP/G$ functional and $LANL2DZ$ Los Alamos double-valence basis set. The energy values of the frontier π-states of the molecule, *i.e.*, the HOMO (Highest Occupied Molecular Orbital) and LUMO (Lowest Unoccupied Molecular Orbital) are tabulated in the Table S5.

6.2.3 Single Point Energy Calculations with Electrodes

Attaching Electrodes: We attached the minimized geometries to two 18-atom Au electrode clusters after manually deleting the terminal thiol's hydrogen atom. The geometries of

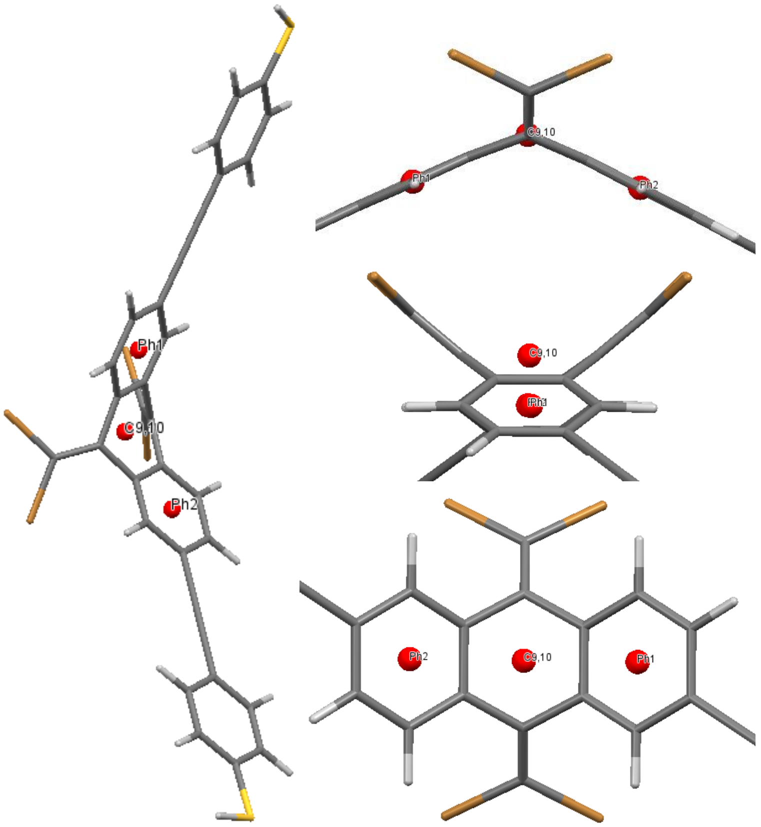

Figure S17: Different views on the points (in red) used to calculate the agle ϕ to estimate the distortion of the core.

the electrode clusters used in these calculations had two layers of 9 atoms each, arranged in a hexagonal close-packed fcc Au-111 surface (see Fig. S18). The Au-Au distance was set to 2.88 Å. S-Au distance was maintained at a value of 2.48 Å and S was attached to the center of the hexagonal close-pack hollow site, taken from literature.[S25] This is all similar to previously reported work.[S26] We kept the geometrical parameters of these electrodes and the electrode material same throughout all these calculations, so that qualitative comparisons could be drawn, while varying the molecule in the junction.

After attaching the molecules to the electrodes, we then calculated the single-point energies using the standard SCF convergence criteria using the *Orca* DFT package. $B3LYP/G$ DFT method was applied and $LANL2DZ$ basis set was used, same as in previous section. Please note, the transmission calculations, described later in section 6.2.4, were not performed on these geometries.

Frontier Molecular Orbitals: From this single-point energy calculation, we obtained the values of E_{HOPS} and E_{LUPS} of the metal-molecule-metal junction, which is tabulated in table S5. We also generated the frontier molecular π-orbitals, HOPS and LUPS, using the VMD and Blender software,[S27] which are shown in figure S18.

Table S5: Table showing the values of E_{HOMO}, E_{LUMO}, E_{HOPS} and E_{LUPS}, the energies of the frontier molecular orbitals in gas-phase optimized geometry and the ideal metal-molecule-metal junctions, calculated in sections 6.2.2 and 6.2.3. All the values are in the units of eV.

	E_{HOMO}	E_{LUMO}	E_{HOPS}	E_{LUPS}
**AQ**	-5.98	-3.24	-6.35	-3.44
**AMe**	-5.44	-1.72	-5.75	-2.03
**APh**	-5.48	-1.87	-5.74	-2.17
**A(CH_2)**	-5.62	-2.05	-5.89	-2.33
**A(All)**	-5.44	-1.92	-5.80	-2.25
**A(Alk)**	-5.60	-2.79	-5.85	-2.32
**ABr**	-5.80	-2.35	-6.24	-2.70
**ATTF**	-4.86	-1.91	-5.12	-2.21
**TCNAQ**	-6.19	-3.99	-6.45	-4.19
**AF**	-5.77	-2.21	-6.21	-2.44

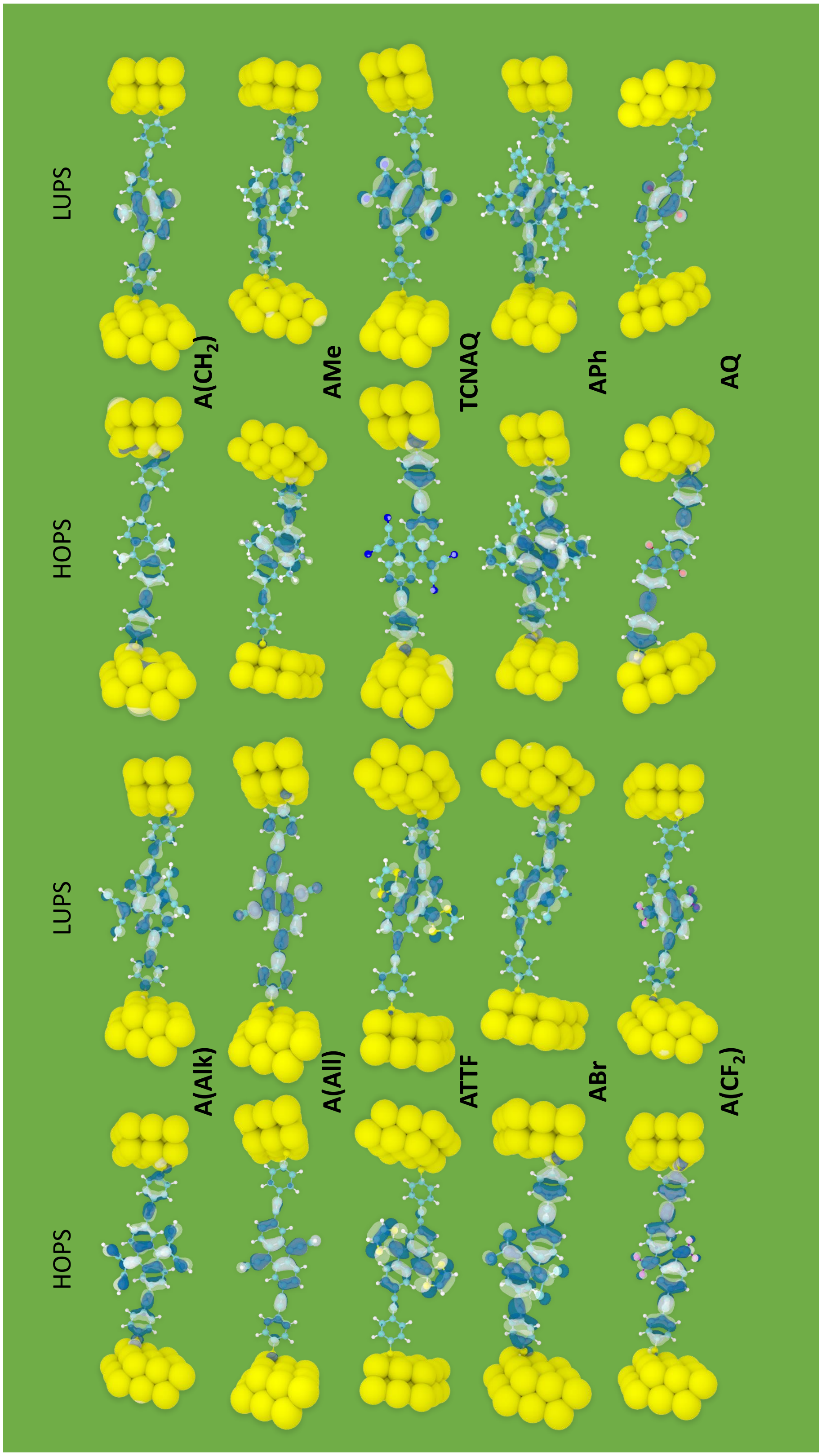

Figure S18: Frontier Molecular Orbitals in the ideal metal-molecule-metal junctions; Highest Occupied π-State (HOPS) and Lowest Unoccupied π-State (LUPS) for the molecular series studied in this paper.

6.2.4 Transport Properties

For computing the electron transmission probability as a function of the energy of the electron, we ran single point energy calculations on molecules without Au electrode clusters and terminating with S atoms (*i.e.*, without H atoms on terminal thiols), using the same *Orca* parameters as in section 6.2.3. We extracted the Hamiltonian (Fock) and overlap matrices from the output of these single-point energy calculations with the commands 'Print[P_Iter_F] 1', 'Print[P_Overlap] 1', 'Print[P_Mos] 1', 'Print[P_InputFile] 1' in the '%output' section. Further, in the artaios input parameter file,[S22,S23] the two terminal S atoms in all the diradical molecules served as the left and right leads, while the rest of the molecule served as the central moiety. Thus, we effectively calculated the transmission probability of the electron traveling across the molecular backbone, without the Au metal clusters.

The transmission probability of the compounds that we were able to synthesize and measure experimentally are summarized in Figure S19.

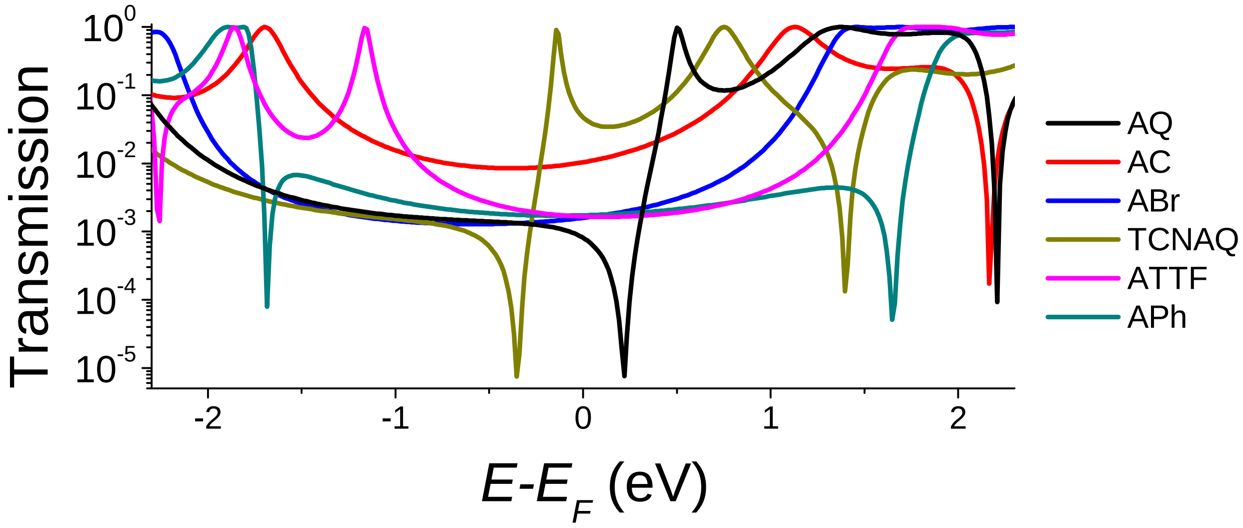

Figure S19: Transmission Probability of the anthraquinoid molecular wires measured on the experimental platforms versus energy of the electron referenced to a E_f value of $-4.3\,\mathrm{eV}$.

Further, to show that the bending of the core of the molecular wires does not switch the QI feature on and off or affect the transmission across the molecular junction, we bent the core of the planar **AQ** molecule. The angle by which the molecular wire was bent *in*

silico was same as the angle of the **APh** molecular wire (47°). As can be seen in figure S20, the transmission across the energy band remains quite the same except for the interference feature which moves higher in the energy by $\sim 250\,\text{meV}$. Similar considerations can be made for **A(All)** for which the bending of the core also affect the transport only slightly. More importantly, it does not reduce this case to that of the other all-carbon derivatives showing that the differences in transport between **A(All)** and **A(CH$_2$)** come from the different electronic structures rather than purely geometrical considerations (figure S21).

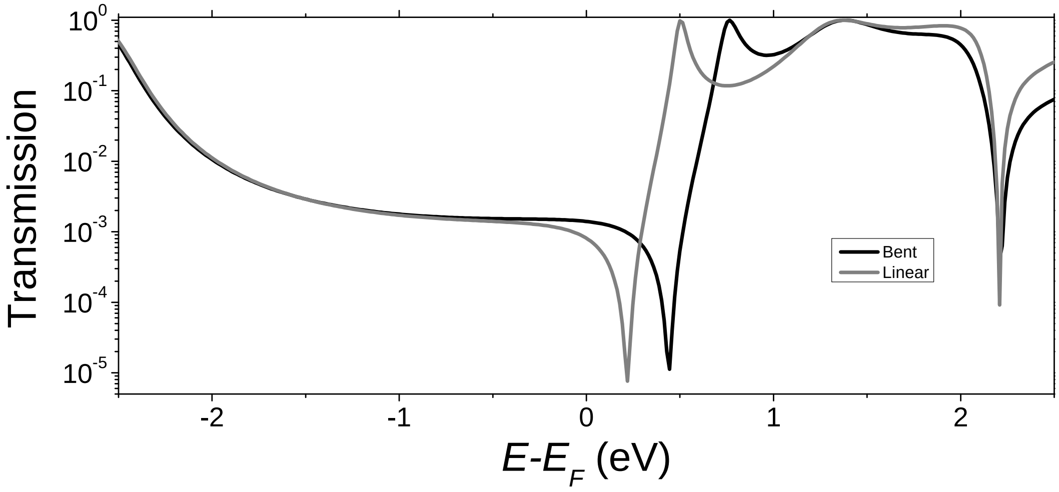

Figure S20: Transmission Probability of the anthraquinone molecular wire in the linear (DFT optimized structure) and bent form. The angle by which the anthraquinone molecular wire was bent is 47° which is same as **APh**. The value of E_{F} used here to reference the energy axis is $-4.3\,\text{eV}$.

Estimation of E_{F}: The transmission spectra obtained from the *artaios* calculations give the transmission probability versus the absolute electron energy referenced to the vacuum level. To make the qualitative analysis of the position of the resonance peaks corresponding to the frontier orbitals and the QI dips in the transmission spectra, it is necessary to align the electron energy axis (x-axis) with respect to the energy of the Fermi level of the bulk electrode material (E_{F}). It is non-trivial to find a reasonable value of E_{F} because of the limitations of DFT calculations. In our previous works, we have used techniques like transition voltage spectroscopy or experimental cyclic voltammetry data to find a E_{F} value as reference, starting

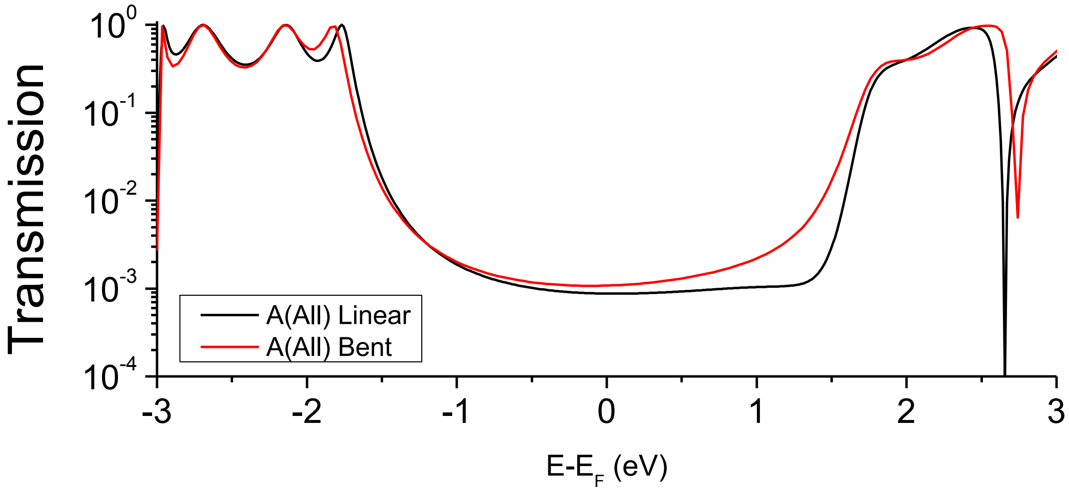

Figure S21: Transmission Probability of **A(All)** in the linear (DFT optimized structure) and bent form. The angle by which the anthraquinone molecular wire was bent is 35° which is same as $\mathbf{A(CH_2)}$. The value of E_F used here to reference the energy axis is −4.3 eV.

from the frontier energy levels.[S6,S7] The same methodology can not be applied in this work because the inherent dissimilar and unique properties of the molecular wires measured in this work. For instance, using transition voltage values for the series doesn't work because the differential conductance curves have different line shapes, and therefore, the obtained transition voltage parameters may or may not refer to the same electronic properties of the individual molecules. (For example, the transition voltage values for **AQ** is smaller than **TCNAQ**, *i.e.*, the LUMO of **AQ** should be lower, however, according to CV the LUMO of **AQ** is higher than **TCNAQ**.)

We used the E_F of −4.3 eV to scale the energy axis in all the transmission curve graphs. It is known that the E_F of EGaIn electrode is about −4.3 eV.[S28] And also, this value of E_F for Au is also supported by several already reported UPS measurements where it has been established experimentally that aliphatic and conjugated SAMs on Au substrates shift the E_F values of Au by 0.85 and 0.98 eV, respectively (*i.e.*, from E_F =−5.2 eV for a clean gold surface to E_F = −4.2 eV to −4.4 eV for Au covered with SAMs).[S29–S31] Thus using E_F value of −4.3 eV makes it more realistic to make qualitative comparisons between the trends in transmission calculations with the trends in experimental measurements on Au/SAM//EGaIn

platforms. This methodology is same as our previously published works.[S7]

7 Chemical Characterization

7.1 ^{1}H and ^{1}3C NMR Spectroscopy

Included below are the ^{1}H and ^{13}C NMR spectra with structures included for **AMe**, **ATTF**, **ABr** and **APh**.

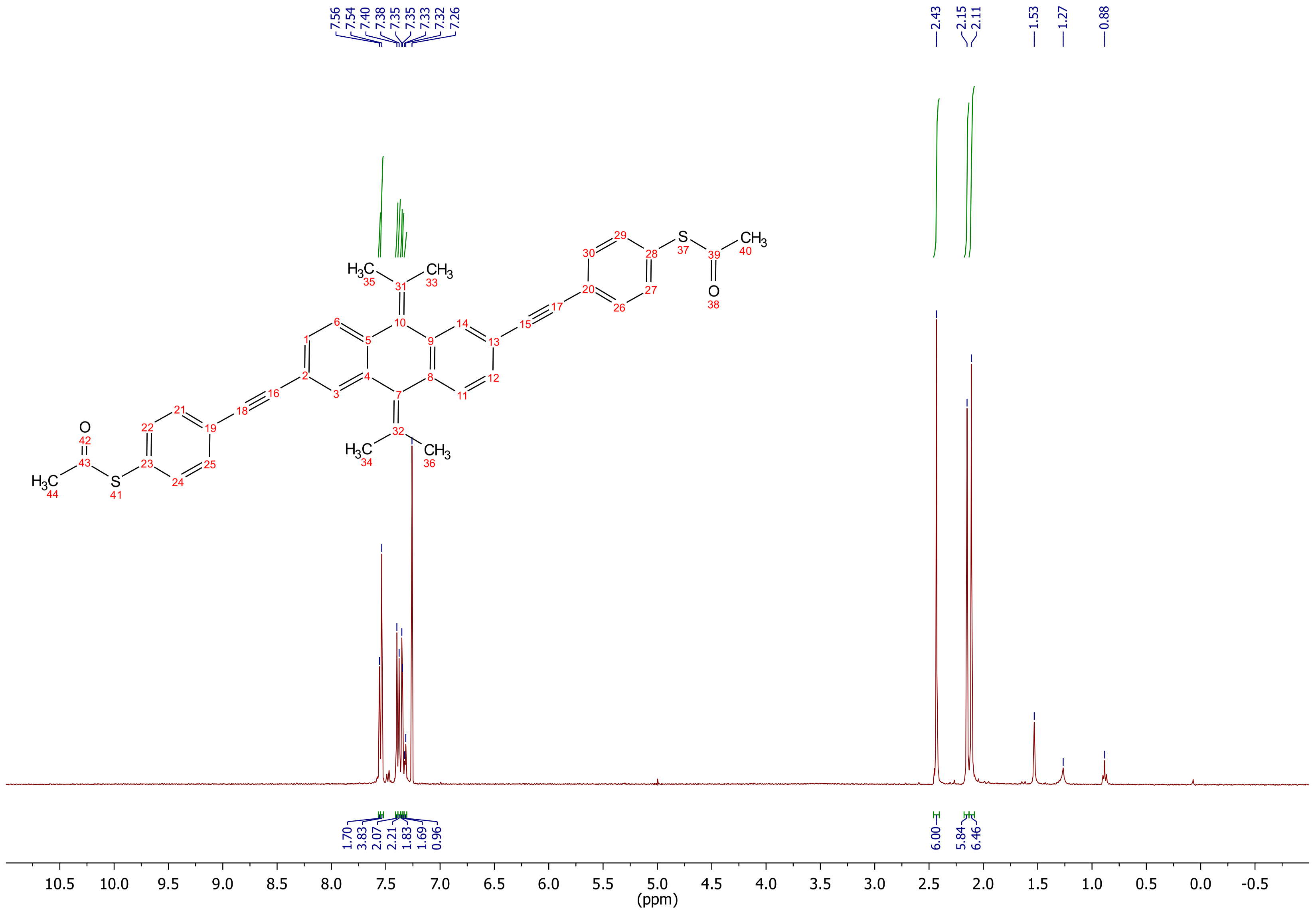
7.56
7.54
7.40
7.38
7.35
7.35
7.33
7.32
7.26
2.43
2.15
2.11
1.53
1.27
0.88
1.70
3.83
2.07
2.21
1.83
1.69
0.96
6.00
5.84
6.46
(ppm)

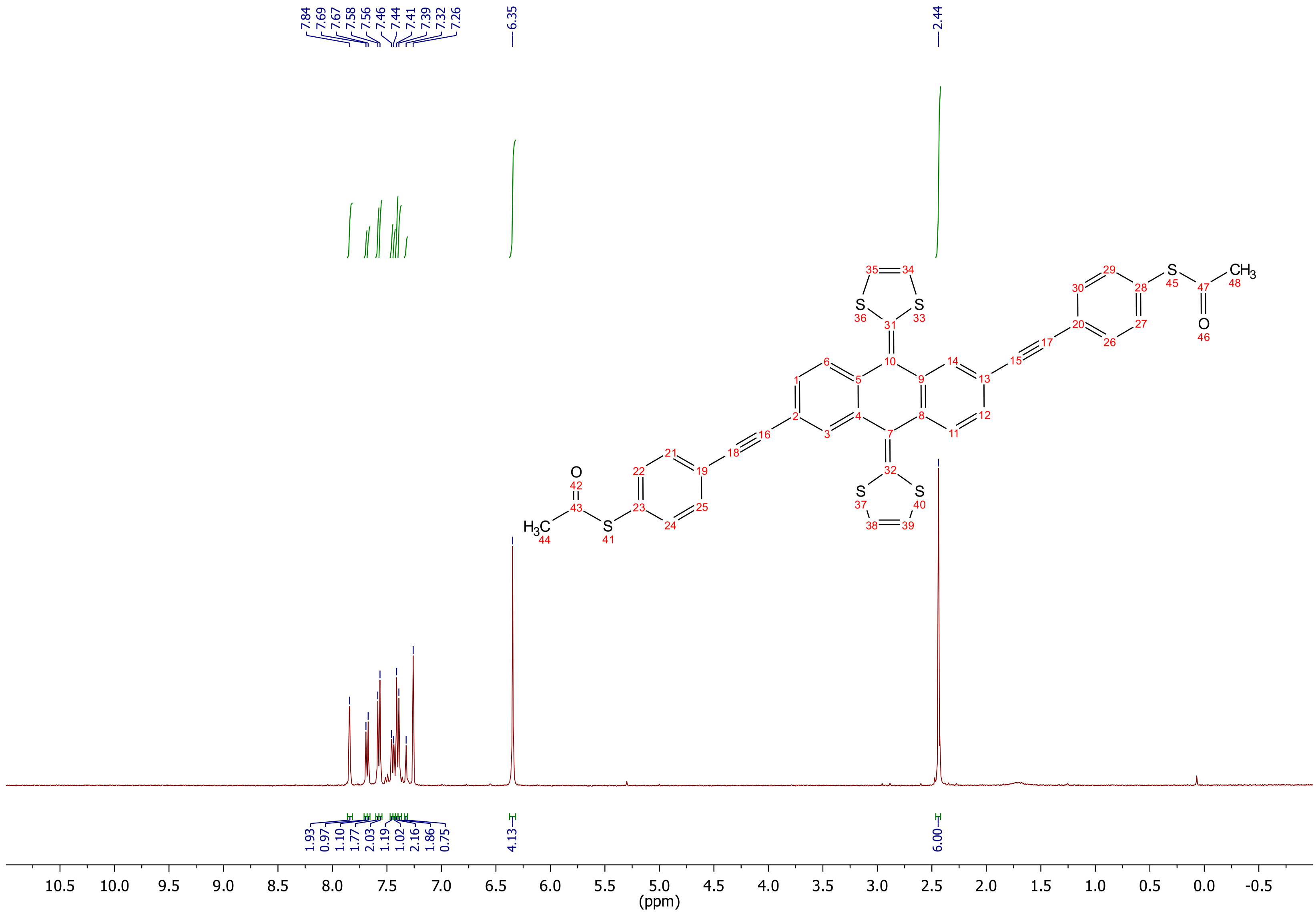

7.84
7.69
7.67
7.58
7.56
7.46
7.44
7.41
7.39
7.32
7.26
6.35
2.44
1.93
0.97
1.10
1.77
2.03
1.19
1.02
2.16
1.86
0.75
4.13
6.00
(ppm)

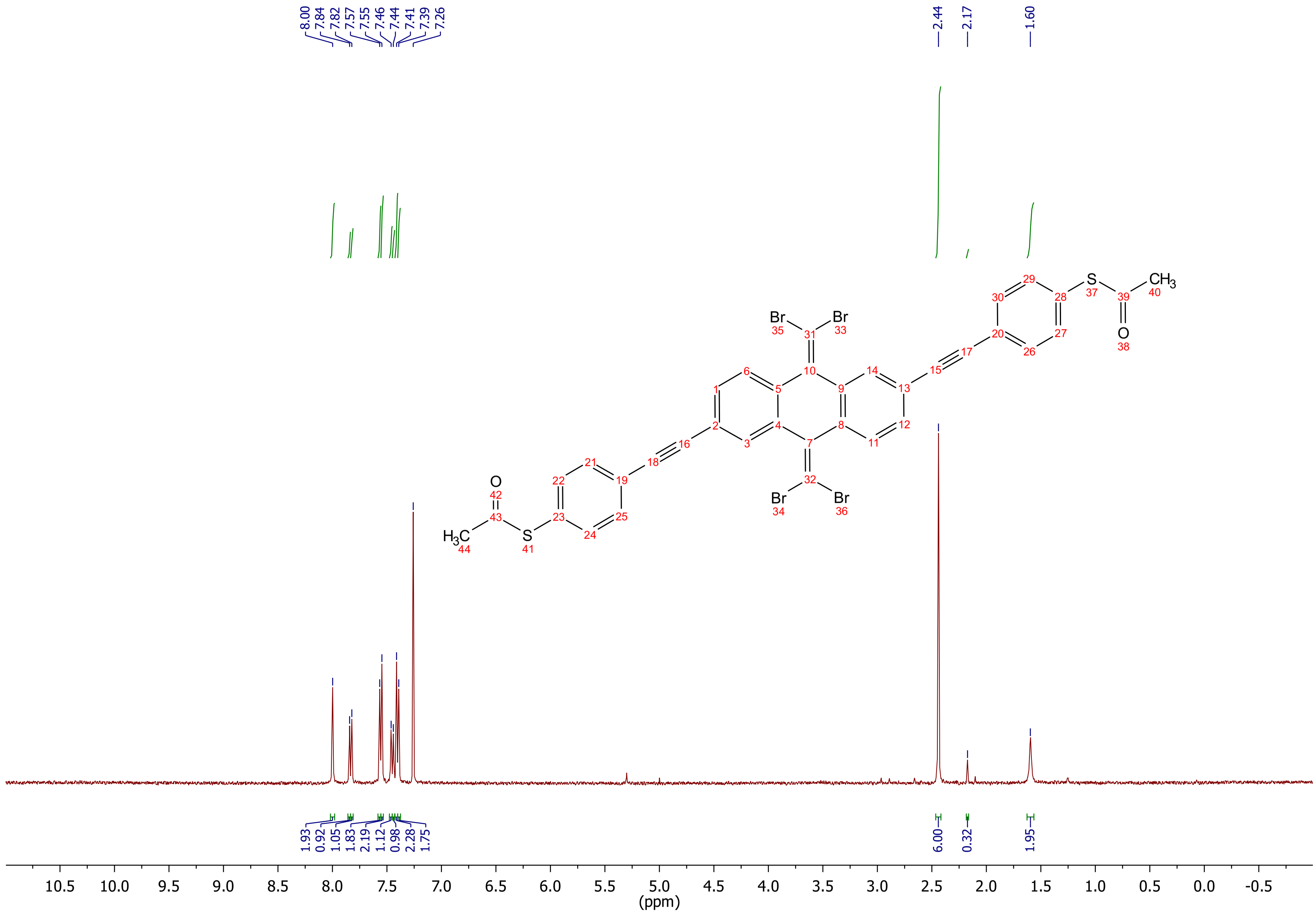

8.00
7.84
7.82
7.57
7.55
7.46
7.44
7.41
7.39
7.26
2.44
2.17
1.60
1.93
0.92
1.05
1.83
2.19
1.12
0.98
2.28
1.75
6.00
0.32
1.95
10.5
10.0
9.5
9.0
8.5
8.0
7.5
7.0
6.5
6.0
5.5
5.0
4.5
4.0
3.5
3.0
2.5
2.0
1.5
1.0
0.5
0.0
-0.5
(ppm)

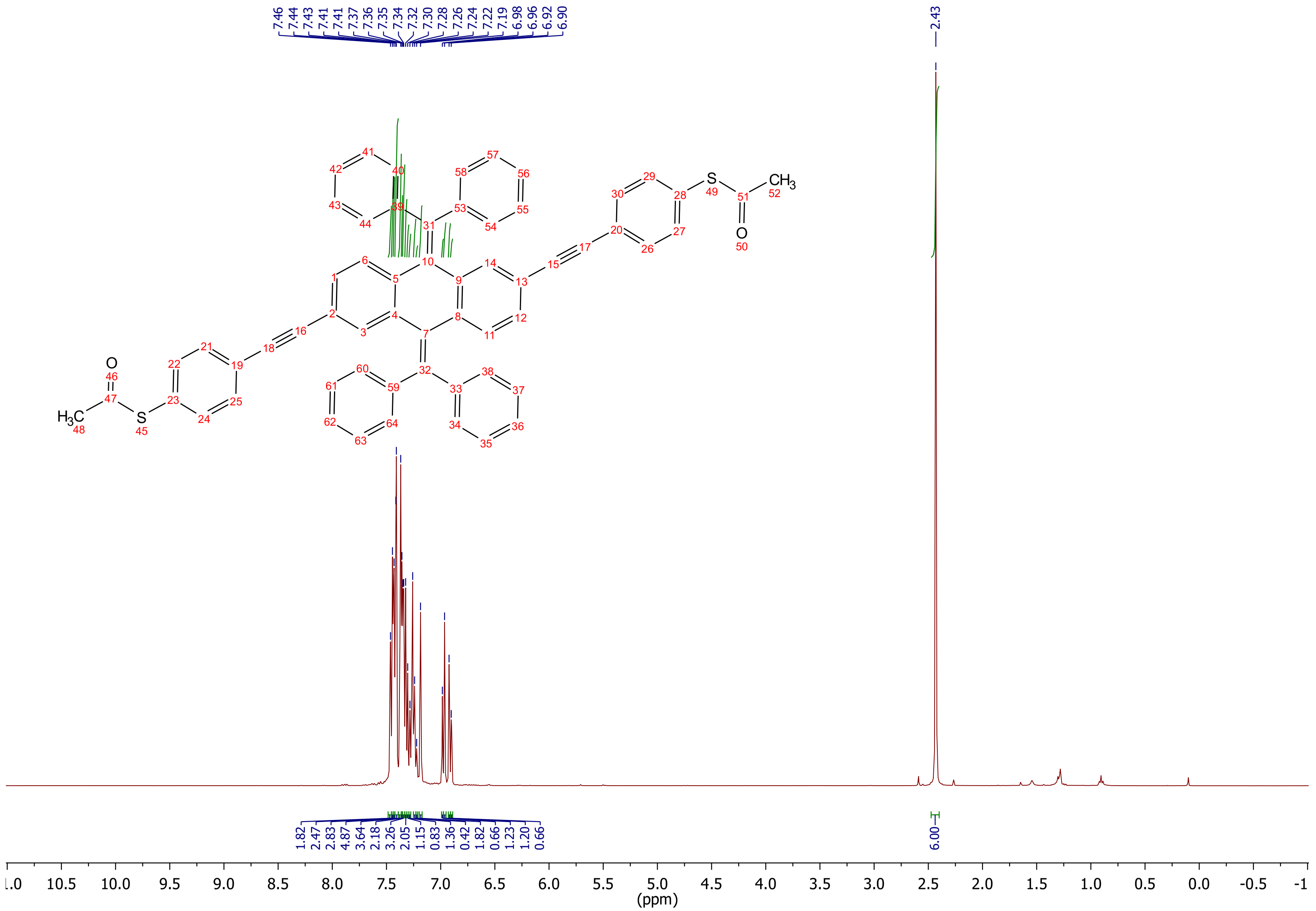
7.46
7.44
7.43
7.41
7.41
7.37
7.36
7.35
7.34
7.32
7.30
7.28
7.26
7.24
7.22
7.19
6.98
6.96
6.92
6.90
2.43
H3C
S
O
CH3
1.82
2.47
2.83
4.87
3.64
2.18
3.26
2.05
1.15
0.83
1.36
0.42
1.82
0.66
1.23
1.20
0.66
6.00
(ppm)

References

(S1) Fracasso, D.; Valkenier, H.; Hummelen, J. C.; Solomon, G. C.; Chiechi, R. C. Evidence for Quantum Interference in SAMs of Arylethynylene Thiolates in Tunneling Junctions With Eutectic Ga–In (EGaIn) Top-Contacts. *J. Am. Chem. Soc.* **2011**, *133*, 9556–9563.

(S2) Carlotti, M.; Soni, S.; Kumar, S.; Ai, Y.; Sauter, E.; Zharnikov, M.; Chiechi, R. C. Two-Terminal Molecular Memory via Reversible Switching of Quantum Interference Features in Tunneling Junctions. *Angew. Chem. Int. Ed.* **2018**, *57*, 15681–15685.

(S3) Valkenier, H.; Guedon, C. M.; Markussen, T.; Thygesen, K. S.; van der Molen, S. J.; Hummelen, J. C. Cross-Conjugation and Quantum Interference: A General Correlation? *Phys. Chem. Chem. Phys.* **2014**, *16*, 653–662.

(S4) Glatzhofer, D. T.; Liang, Y.; Khan, M. A. Formation, structure, and reactivity of dibenzo-p-quinodimethane stabilized as .eta.6-ligands of (cyclopentadienyl)- and (pentamethylcyclopentadienyl)ruthenium cations. *Organometallics* **1993**, *12*, 624–632.

(S5) Carlotti, M.; Degen, M.; Zhang, Y.; Chiechi, R. C. Pronounced Environmental Effects on Injection Currents in EGaIn Tunneling Junctions Comprising Self-Assembled Monolayers. *J. Phys. Chem. C* **2016**, *120*, 20437–20445.

(S6) Carlotti, M.; Kovalchuk, A.; Wächter, T.; Qiu, X.; Zharnikov, M.; Chiechi, R. C. Conformation-Driven Quantum Interference Effects Mediated by Through-Space Conjugation in Self-Assembled Monolayers. *Nat. Commun.* **2016**, *7*, 13904.

(S7) Zhang, Y.; Ye, G.; Soni, S.; Qiu, X.; Krijger, T. L.; Jonkman, H. T.; Carlotti, M.; Sauter, E.; Zharnikov, M.; Chiechi, R. C. Controlling destructive quantum interference in tunneling junctions comprising self-assembled monolayers via bond topology and functional groups. *Chem. Sci.* **2018**, *9*, 4414–4423.

(S8) Tai, Y.; Shaporenko, A.; Rong, H.-T.; Buck, M.; Eck, W.; Grunze, M.; Zharnikov, M. Fabrication of Thiol-Terminated Surfaces Using Aromatic Self-Assembled Monolayers. *J. Phys. Chem. B* **2004**, *108*, 16806–16810.

(S9) Zharnikov, M. High-Resolution X-Ray Photoelectron Spectroscopy in Studies of Self-Assembled Organic Monolayers. *J. Electron Spectrosc. Relat. Phenom.* **2010**, *178–179*, 380–393.

(S10) Schreiber, F. Structure and Growth of Self-Assembling Monolayers. *Prog. Surf. Sci.* **2000**, *65*, 151–257.

(S11) Aitchison, H.; Lu, H.; Hogan, S. W. L.; Früchtl, H.; Cebula, I.; Zharnikov, M.; Buck, M. Self-Assembled Monolayers of Oligophenylenecarboxylic Acids on Silver Formed at the Liquid–Solid Interface. *Langmuir* **2016**, *32*, 9397–9409.

(S12) Stohr, J. *NEXAFS Spectroscopy*; Springer-Verlag Berlin Heidelberg, 1992.

(S13) Horsley, J. A.; Stöhr, J.; Hitchcock, A. P.; Newbury, D. C.; Johnson, A. L.; Sette, F. Resonances in the K shell excitation spectra of benzene and pyridine: Gas phase, solid, and chemisorbed states. *The Journal of Chemical Physics* **1985**, *83*, 6099–6107.

(S14) Yokoyama, T.; Seki, K.; Morisada, I.; Edamatsu, K.; Ohta, T. X-ray absorption spectra of poly- p -phenylenes and polyacenes: localization of π orbitals. *Physica Scripta* **1990**, *41*, 189–.

(S15) Hamoudi, H.; Kao, P.; Nefedov, A.; Allara, D. L.; Zharnikov, M. X-Ray Spectroscopy Characterization of Self-Assembled Monolayers of Nitrile-Substituted Oligo(phenylene Ethynylene)s With Variable Chain Length. *Beilstein J. Nanotechnol.* **2012**, *3*, 12–24.

(S16) Frey, S.; Stadler, V.; Heister, K.; Eck, W.; Zharnikov, M.; Grunze, M.; Zeysing, B.; Terfort, A. Structure of Thioaromatic Self-Assembled Monolayers on Gold and Silver. *Langmuir* **2001**, *17*, 2408–2415.

(S17) Shaporenko, A.; Adlkofer, K.; Johansson, L.; Tanaka, M. Functionalization of GaAs Surfaces With Aromatic Self-Assembled Monolayers: A Synchrotron-Based Spectroscopic Study. *Langmuir* **2003**, *19*, 4992–4998.

(S18) Zhao, A.; Tan, S.; Li, B.; Wang, B.; Yang, J.; Hou, J. G. STM tip-assisted single molecule chemistry. *Phys. Chem. Chem. Phys.* **2013**, *15*, 12428–12441.

(S19) Valkenier, H.; Huisman, E. H.; van Hal, P. A.; de Leeuw, D. M.; Chiechi, R. C.; Hummelen, J. C. Formation of High-Quality Self-Assembled Monolayers of Conjugated Dithiols on Gold: Base Matters. *J. Am. Chem. Soc.* **2011**, *133*, 4930–4939.

(S20) Neese, F. The ORCA Program System. *Wiley Interdiscip. Rev.: Comput. Mol. Sci.* **2012**, *2*, 73–78.

(S21) Neese, F. Software update: the ORCA program system, version 4.0. *Wiley Interdisciplinary Reviews: Computational Molecular Science*

(S22) Herrmann, C.; Gross, L.; Steenbock, T.; Deffner, M.; Voigt, B. A.; Solomon, G. C. ARTAIOS - A Transport Code for Postprocessing Quantum Chemical Electronic Structure Calculations, Available From https://www.chemie.uni-hamburg.de/ac/herrmann/software/index.html. 2016.

(S23) Herrmann, C.; Solomon, G. C.; Subotnik, J. E.; Mujica, V.; Ratner, M. A. Ghost Transmission: How Large Basis Sets Can Make Electron Transport Calculations Worse. *J. Chem. Phys.* **2010**, *132*, 024103.

(S24) Weigend, F.; Ahlrichs, R. Balanced Basis Sets of Split Valence{,} Triple Zeta Valence and Quadruple Zeta Valence Quality for H to Rn: Design and Assessment of Accuracy. *Phys. Chem. Chem. Phys.* **2005**, *7*, 3297–3305.

(S25) Bilić, A.; Reimers, J. R.; Hush, N. S. The structure, energetics, and nature of the chemical bonding of phenylthiol adsorbed on the Au(111) surface: Implications for density-

functional calculations of molecular-electronic conduction. *The Journal of Chemical Physics* **2005**, *122*, 094708.

(S26) Schlicke, H.; Herrmann, C. Controlling Molecular Conductance: Switching Off π Sites Through Protonation. *ChemPhysChem* **2014**, *15*, 4011–4018.

(S27) Humphrey, W.; Dalke, A.; Schulten, K. VMD – Visual Molecular Dynamics. *Journal of Molecular Graphics* **1996**, *14*, 33–38.

(S28) Nerngchamnong, N.; Yuan, L.; Qi, D.-C.; Li, J.; Thompson, D.; Nijhuis, C. a. The Role of Van Der Waals Forces in the Performance of Molecular Diodes. *Nat. Nanotechnol.* **2013**, *8*, 113–118.

(S29) Cabarcos, O. M.; Schuster, S.; Hehn, I.; Zhang, P. P.; Maitani, M. M.; Sullivan, N.; Giguère, J.-B.; Morin, J.-F.; Weiss, P. S.; Zojer, E.; Zharnikov, M.; Allara, D. L. Effects of Embedded Dipole Layers on Electrostatic Properties of Alkanethiolate Self-Assembled Monolayers. *The Journal of Physical Chemistry C* **2017**, *121*, 15815–15830.

(S30) Abu-Husein, T.; Schuster, S.; Egger, D. A.; Kind, M.; Santowski, T.; Wiesner, A.; Chiechi, R. C.; Zojer, E.; Terfort, A.; Zharnikov, M. The Effects of Embedded Dipoles in Aromatic Self-Assembled Monolayers. *Adv. Funct. Mater.* **2015**, *25*, 3943–3957.

(S31) Kovalchuk, A.; Abu-Husein, T.; Fracasso, D.; Egger, D. A.; Zojer, E.; Zharnikov, M.; Terfort, A.; Chiechi, R. C. Transition Voltages Respond to Synthetic Reorientation of Embedded Dipoles in Self-Assembled Monolayers. *Chem. Sci.* **2016**, *7*, 781–787.
